# Glycerol-driven energy and proteostasis underpin antibiotic tolerance in *Escherichia coli*

**DOI:** 10.1016/j.isci.2026.116896

**Published:** 2026-07-22

**Authors:** Han G. Ngo, Sayed Golam Mohiuddin, Mehmet A. Orman

**Affiliations:** 1 Department of Chemical and Biomolecular Engineering, University of Houston, Houston, TX 77204, USA; 2 Department of Biomedical Engineering, University of Wisconsin-Madison, Madison, WI 53706, USA

**Keywords:** persister, *Escherichia coli*, metabolism, TCA cycle, protein aggregation, large polar aggregates, phospholipid recycling

## Abstract

The metabolic pathways that sustain bacterial persistence during nutrient limitation remain poorly understood. Using stationary-phase *Escherichia coli* as a model of antibiotic tolerance, we used proteomics, genetics, metabolic phenotyping, and single-cell imaging to define a metabolic framework underlying persistence. Perturbation of tricarboxylic acid cycle function reprogrammed stationary-phase physiology, suppressing lipid and glycerol metabolism, altering energy homeostasis and proteostasis, and reducing antibiotic tolerance. Systems-level analyses identified phospholipid-derived glycerol catabolism as a critical metabolic pathway linking carbon recycling to persistence. Genetic disruption of key nodes within this pathway impaired proton motive force homeostasis, reduced large polar protein aggregate formation, altered division-associated remodeling, and sensitized cells to antibiotic-induced lysis. Metabolic assays further revealed that persisters retain a selective capacity to utilize glycerol for rapid proton motive force restoration. Together, these findings support a model in which stationary-phase persisters are sustained through metabolic rewiring that coordinates energy maintenance, proteostasis, and antibiotic tolerance.

## Highlights

• Lipid-derived glycerol fuels persistence in late stationary-phase *E. coli*
• Glycerol catabolism sustains basal energy homeostasis in persisters
• Disruption of glycerol catabolism reprograms stress and proteostasis networks
• Large polar aggregates correlate with reduced antibiotic-induced cell lysis

## Graphical abstract

**Figure:**
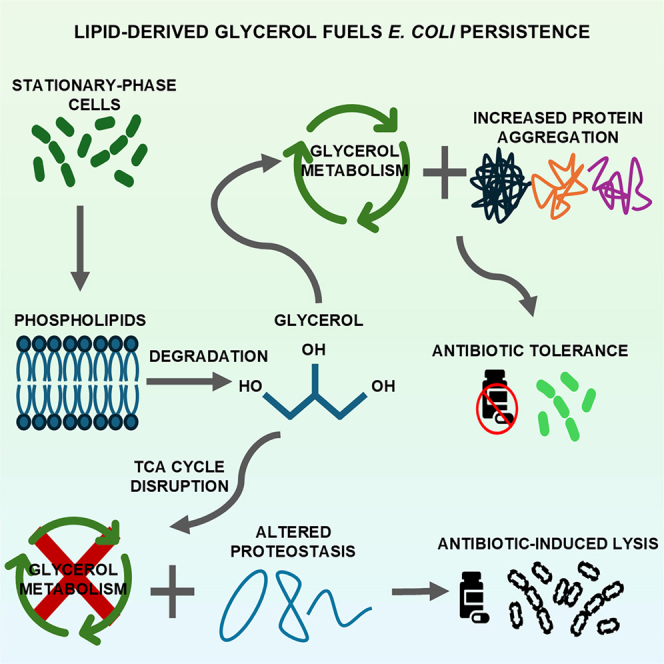

## Teaser

biological sciences; molecular biology; microbiology

## Introduction

Although bacterial persisters have been discovered and studied for over eight decades,^1^^,^^2^ their underlying mechanisms are still elusive. This is largely due to the abundance and complexity of bacterial survival strategies, which involve numerous cellular processes that vary depending on environmental factors, growth and treatment conditions, antibiotic types, and bacterial species.^3^^,^^4^^,^^5^^,^^6^^,^^7^ Persisters have been identified in a wide range of bacteria, including *Escherichia coli* (*E. coli*), *Mycobacterium tuberculosis*, and *Staphylococcus aureus*, as well as in fungi and even cancer cells.^3^^,^^4^^,^^5^^,^^6^^,^^7^^,^^8^^,^^9^^,^^10^^,^^11^ These cells are capable of surviving prolonged and intensive antibiotic or chemotherapeutic treatments without acquiring genetic mutations that induce resistance.^12^ Persisters are thought to be one of the reasons why antibiotic treatment failure, relapse of chronic infections, and the emergence of antibiotic resistance are happening.^13^^,^^14^^,^^15^^,^^16^ Despite significant progress, a comprehensive understanding of the diverse and context-dependent mechanisms of persistence is still needed to guide the development of effective anti-persister therapies.

Persister cells are frequently characterized as metabolically dormant. While this description captures an important aspect of their physiology (i.e., reduced anabolism and proliferation relative to exponentially growing cells), they should retain a basal level of metabolic activity and energy production.^17^^,^^18^^,^^19^^,^^20^ This basal metabolism may support key cellular functions such as resuming growth upon antibiotic removal,^21^ initiating stress responses like the DNA damage response (SOS) pathway,^22^ repairing DNA damage,^23^ sustaining energy through possible futile cycles,^6^ and utilizing specific carbon sources that potentiate aminoglycoside (AG) killing,^24^ all of which indicate a degree of physiological robustness in persisters. Indeed, more recent studies reveal that persister cells are highly metabolically heterogeneous^25^ and can dynamically reprogram gene expression and regulatory networks to survive antibiotic stress.^26^

Our previous work demonstrated that cyclic adenosine monophosphate (cAMP), together with its receptor protein, cAMP receptor protein (CRP), functions as a key metabolic regulator during the stationary phase, shifting cellular metabolism from anabolic processes toward oxidative phosphorylation.^27^ This metabolic transition supports a basal level of energy production, which may be required for the survival of persister cells formed under these conditions.^27^ Consistent with this idea, genetic disruption of these regulatory and metabolic pathways significantly reduced persistence, particularly against ampicillin and ofloxacin.^27^ For instance, in our *E. coli* Keio knockout screen, we observed the most substantial reductions in persister levels among strains lacking key genes, always involved in the tricarboxylic acid (TCA) cycle, electron transport chain (ETC), and adenosine triphosphate (ATP) synthase, while a few gene deletions in TCA and ETC components (such as *icd*, *nuoL*) led to modest increases in persistence.^27^ While these findings were further validated in independently constructed *E. coli* MG1655 deletion strains,^27^ the factors mediating these pathways in the stationary phase, particularly carbon sources or intracellular recycling, remain to be determined.

Bacterial cells in the stationary phase, particularly in nonnutritive environments, initiate intracellular self-digestion by degrading their own components, such as proteins and lipids, which serves as a survival strategy under nutrient-limiting conditions.^28^^,^^29^^,^^30^^,^^31^ Our previous work showed that this intracellular degradation correlates with persistence, likely by supplying essential metabolites and energy precursors,^32^ as we found that inhibiting stationary-phase metabolism simultaneously reduces both intracellular degradation and persistence.^32^ In eukaryotes, autophagy is a well-established mechanism for maintaining viability under nutrient deprivation and has been directly linked to drug tolerance. By contrast, analogous processes of self-digestion and intracellular recycling in bacteria remain far less well defined. In particular, the specific metabolic pathways and molecular players, including preferred carbon sources, driving this self-sustaining metabolic state remain largely uncharacterized. Resolving this knowledge gap is essential to understanding the metabolic underpinnings of antibiotic tolerance. In this study, we began with proteomic analysis of the succinate dehydrogenase knockout (Δ*sdhA*), a strain exhibiting markedly reduced stationary-phase persisters,^27^ to pinpoint metabolic pathways most disrupted in the stationary phase when persistence is lost. We systematically investigated the role of these metabolic components in shaping persister physiology and found that stationary-phase *E. coli* can recycle lipids to fuel glycerol metabolism, maintain energy, and survive antibiotics.

## Results

### SdhA deletion rewires stress, redox, and metabolic pathways in stationary-phase *E. coli*

**Figure 1:**
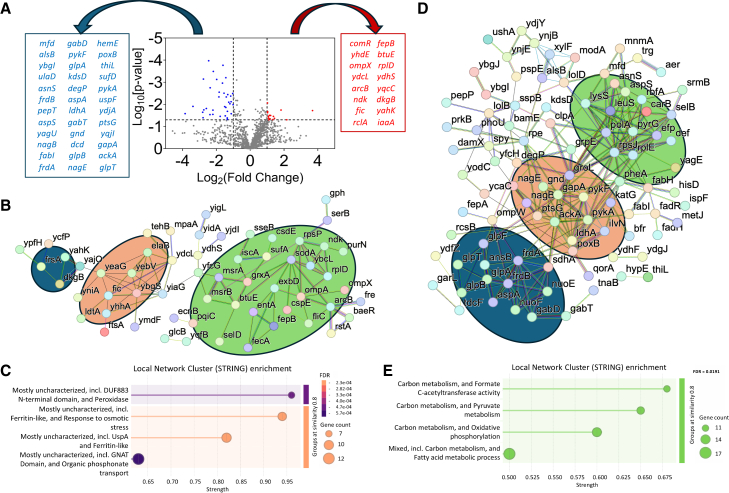
Proteomic analysis reveals broad metabolic and stress-response remodeling in the Δ*sdhA* strain during late stationary phase (A) Volcano plot showing significantly upregulated (red) and downregulated (blue) proteins in Δ*sdhA* relative to WT *E. coli* K-12 MG1655 collected at late stationary phase (24 h). The fold-change thresholds were set at ≤ −2 and ≥2, with a *p* value cutoff of 0.05. (B) Pathway enrichment analysis of upregulated protein networks using STRING.^35^ Observed clusters are highlighted with colored circles: blue, detoxification and redox balance; orange, stringent response and stationary-phase adaptation; green, oxidative stress response, iron homeostasis, and global stress signaling. (C) Local network cluster enrichment (STRING) for the upregulated protein sets. (D) Pathway enrichment analysis of downregulated protein networks using STRING.^35^ Observed clusters are highlighted with colored circles: blue, oxidative phosphorylation, glycerol metabolism, and electron transport; orange, central carbon metabolism including glycolysis and pyruvate processing; green, translation and protein synthesis. (E) Local network cluster enrichment (STRING) for the downregulated protein sets. STRING networks are shown in evidence mode, where edges represent predicted protein-protein associations supported by experimental and computational evidence. Local network clusters include false discovery rate (FDR; Benjamini-Hochberg-adjusted *p* values) and enrichment strength [log10(observed/expected)]. Three independent biological replicates were analyzed (*N* = 3). See also Figure S3 and Table S1.

To investigate the physiological consequences of energy metabolism disruption in persister formation, we performed untargeted proteomic analysis on the Δ*sdhA* mutant during the late stationary phase. To reach this phase, overnight cultures were diluted into Luria-Bertani (LB) medium and grown for 24 h, following the same experimental strategy used in our previous studies.^27^^,^^32^ This stage is critical for persistence, as cells typically transition into a low-metabolic-activity, stress-adapted state that promotes survival under antibiotic exposure. Δ*sdhA* was chosen because it represents a central node in both the TCA cycle and ETC, and consistently yields fewer persisters when transferred from stationary phase to fresh medium under antibiotic treatment.^27^^,^^33^ We first assessed global protein abundance in the Δ*sdhA* knockout and compared it to the wild-type (WT) strain using a volcano plot (Figure 1A, and data and code availability), which illustrates fold-changes in protein expression. Statistical significance was assessed using a parametric *t* test, with an adjusted *p* value cutoff of 0.05.^34^ The functions of significantly upregulated and downregulated proteins are listed in Table S1. Proteins showing at least a 2-fold increase or decrease in abundance were then analyzed using Search Tool for Retrieval of Interacting Genes/Proteins (STRING)-based network and pathway enrichment tools (Figures 1B–1E), incorporating curated databases such as Gene Ontology, to Encyclopedia of Genes and Genomes (KEGG) pathways, and UniProt functional terms.^35^ Enrichment strength was calculated as the log_10_ ratio observed to expected proteins per pathway, and significance was determined using a false discovery rate-corrected *p* value threshold.^35^

The network of upregulated proteins in Δ*sdhA* (Figures 1B and 1C) revealed three major functional clusters associated with (1) detoxification and redox balance, (2) stringent response and stationary-phase adaptation, and (3) oxidative stress response, iron homeostasis, and global stress signaling. The first group included oxidoreductases such as YahK and DkgB, which are involved in detoxifying reactive aldehydes and methylglyoxal, as well as YajO, a thiamine biosynthesis enzyme (the blue cluster, Figure 1B). These proteins likely mitigate damage from metabolic byproducts and contribute to maintaining redox homeostasis.^36^ The second cluster comprised proteins associated with the stringent response and stress adaptation, including YeaG, Fic, ElaB, YiaG, and others (the orange cluster, Figure 1B). These proteins are known to be induced under nutrient limitation and stress and are often regulated by the alarmone ppGpp.^37^ For example, Fic is a stationary-phase regulatory protein involved in energy sensing, and ElaB contributes to survival under oxidative and membrane stress.^38^^,^^39^ The third and most extensive group of upregulated proteins consisted of enzymes and regulators involved in oxidative stress defense (MsrA, MsrB, BtuE, GrxA, SodA, Fre), iron-sulfur cluster assembly (IscA, SufA), and iron transport (EntA, FepB, FecA, ExbD) (the green cluster, Figure 1B). Expression of these proteins indicates a broad cellular response to oxidative burden and iron limitation, conditions typical of the stationary phase, and interestingly, they are significantly elevated in Δ*sdhA*. Although one might expect Δ*sdhA* to reduce oxidative stress, the upregulation of these defense mechanisms may play a protective role, potentially contributing to the maintenance of proteostasis. Also, several components of the ribosome (RpsB, RplD), amino acid metabolism (SerB), and nucleotide balance (Ndk) were upregulated (Figures 1B and 1C), possibly reflecting the need to maintain core metabolic functions despite energy stress. Additionally, regulators such as ArcB, BaeR, and RstA, which mediate anaerobic and envelope stress responses,^40^^,^^41^^,^^42^ were more abundant, suggesting transcriptional reprogramming in response to metabolic disruption.

In contrast, the set of downregulated proteins (Figures 1D and 1E) pointed to a repression of core cellular functions in Δ*sdhA*. We identified three major clusters of functionally related proteins associated with (1) translation and protein synthesis, (2) central carbon metabolism including glycolysis and pyruvate processing, and (3) oxidative phosphorylation, lipid degradation, and glycerol metabolism. Reduced abundance of ribosomal proteins (RpsJ, RplE, MnmA, AsnS, AspS) and translation factors (Efp, SelB, Def) (the green cluster, Figure 1D) suggests a general slowdown in protein synthesis, consistent with energy limitation in stationary-phase cells lacking a functional TCA cycle. Key glycolytic and associated enzymes, including GapA, PykF, Gnd, LdhA, PoxB, and PtsG, were also significantly downregulated (the orange cluster, Figure 1D). These enzymes are responsible for carbon flux through glycolysis and pyruvate metabolism and are tightly linked to energy generation and biosynthetic precursor supply.^37^ Thiamine pyrophosphate (TPP)-dependent enzymes and TPP-binding proteins were especially affected, indicating that decarboxylation steps and cofactor metabolism may be impaired in Δ*sdhA*. Further repression was observed in proteins involved in oxidative phosphorylation and anaerobic respiration (the blue cluster, Figure 1D). In addition to the expected loss of SdhA, components of fumarate and nicotinamide adenine dinucleotide (NADH) dehydrogenase complexes (FrdA, FrdB, NuoE, NuoF) were also decreased. These changes are likely to restrict the cell’s capacity to generate ATP and intermediates necessary for biosynthesis. Multiple proteins involved in glycerol utilization and alternative carbon source metabolism (GlpA, GlpB, GlpF, and YdfZ) were downregulated, suggesting that the mutant is unable to efficiently reroute carbon flux under nutrient-limited conditions. STRING enrichment analysis revealed additional downregulation of pathways such as fatty acid metabolism and formate C-acetyltransferase activity (Figures 1D and 1E), supporting the notion that the energy and biosynthetic flexibility required for stationary-phase survival is compromised in the Δ*sdhA* background. Overall, these results demonstrate that *sdhA* deletion induces broad metabolic and stress-response reprogramming that reshapes stationary-phase physiology, with these alterations further verified in the subsequent sections.

### TCA cycle knockouts do not exhibit altered oxidative damage in the stationary phase

**Figure 2:**
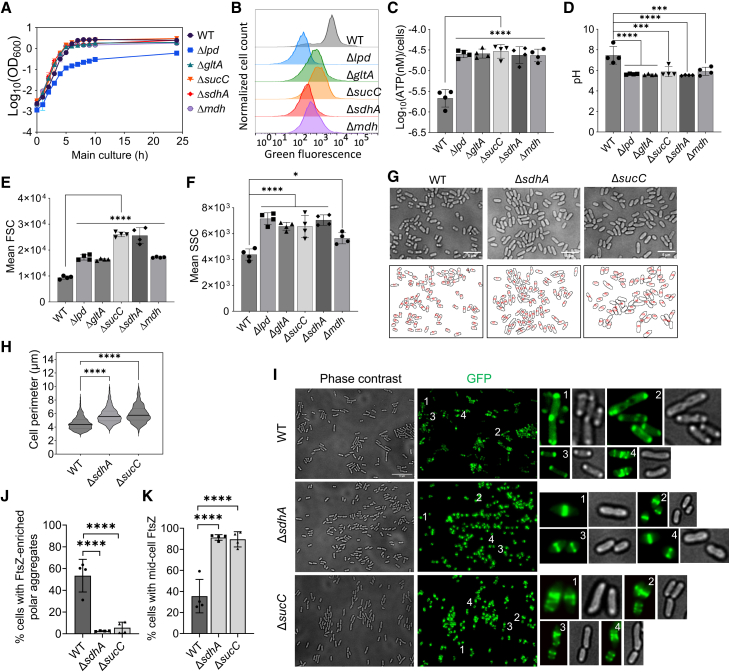
TCA cycle knockout strains exhibit altered redox physiology, increased cell size, and reduced large polar aggregates during late stationary phase (A) Growth curves of wild-type and TCA cycle knockout strains measured by OD_600_ following dilution of overnight cultures into fresh LB medium and incubation for 24 h (*N* = 3). (B) Redox activity of wild-type and mutant strains assessed using Redox Sensor Green (RSG) dye and analyzed by flow cytometry to evaluate respiratory/metabolic activity (*N* = 3). A representative plot is shown; all biological replicates produced similar results. (C) ATP levels measured using the BacTiter-Glo microbial cell viability assay and normalized to cell number (*N* = 4). (D) Intracellular pH levels measured using pGFPR01, which expresses the ratiometric fluorophore pHluorin (*N* = 4). (E and F) Forward scatter (FSC; associated with apparent cell size) and side scatter (SSC; associated with intracellular complexity/granularity) measurements obtained by flow cytometry (*N* = 4). (G) Phase-contrast micrographs of wild-type and mutant strains. A representative image is shown; similar results were observed across four biological replicates (*N* = 4). Scale bars, 5 μm. Cell perimeters were quantified using ImageJ, with segmented micrographs shown below. (H) Quantification of cell perimeters from 1,000 cells per condition. (I) Microscope imaging to visualize structural organization of Z-rings and large polar aggregates in *E. coli* WT, Δ*sdhA*, and Δ*sucC* strains. Representative phase-contrast and corresponding fluorescence images are shown, with selected cells (numbered) enlarged and displayed side by side to highlight diverse morphologies and structural features. (*N* = 4). All biological replicates produced similar results. Scale bars, 10 μm. (J) Quantification of the percentage of cells containing large polar aggregates (LPAs) in *E. coli* WT, Δ*sdhA*, and Δ*sucC* strains. Each dot represents the mean of a biological replicate, based on analysis of thousands of cells across multiple images (*N* = 4). (K) Quantification of the percentage of cells containing Z-rings in *E. coli* WT, Δ*sdhA*, and Δ*sucC* strains. Each dot represents the mean of a biological replicate, based on analysis of thousands of cells across multiple images (*N* = 4). All statistical analyses were performed using one-way ANOVA with Dunnett’s multiple comparisons test. Statistical significance is indicated as follows: ∗*p* < 0.05; ∗∗∗*p* < 0.001; ∗∗∗∗*p* < 0.0001. Data represent the mean ± standard deviation. See also Figures S1, S2, S3, S4, and S5.

Although our proteomic analysis focused on the *ΔsdhA* knockout, the low-persister phenotype and associated metabolic shifts observed in this strain reflect broader physiological trends among other TCA cycle mutants (e.g., *Δlpd*, *ΔgltA*, *ΔsucC*, *Δmdh*), as most of these knockouts generate significantly fewer persisters than WT upon transfer from the stationary phase to fresh medium (thus, these results are not repeated here).^27^^,^^32^ To ensure that these phenotypes were not confounded by major growth abnormalities, we first examined the growth dynamics of the TCA cycle knockouts. Growth curves based on optical density (OD_600_) measurements in LB medium showed that all tested knockouts exhibited growth similar to the WT, except for Δ*lpd* (Figure 2A). Flow cytometry-based cell counts at the late stationary phase (24 h), which provide a more accurate measure of cell abundance, further confirmed that final cell densities were generally comparable across all strains, again with the exception of Δ*lpd* (see Figure S1). While slow growth is thought to be associated with increased persistence, the Δ*lpd* strain challenges this assumption, as it exhibits reduced persistence,^27^ despite its growth defect. Since most mutants do not exhibit growth deficiencies, and all show reduced persistence,^27^ these findings suggest that the low-persister phenotype and downstream phenotypic differences are not attributable to impaired growth or reduced viability in stationary phase. Of note, all follow-up assays were performed using normalized cell numbers to ensure consistency across strains.

Given that many of the proteomic changes in Δ*sdhA* were associated with oxidative stress and redox homeostasis, we next examined whether loss of TCA cycle enzymes results in altered oxidative damage. First, we assessed cell membrane integrity using propidium iodide (PI) staining and flow cytometry. No significant differences in membrane damage were observed between WT and knockout strains at the late stationary phase (see Figure S2). To more directly evaluate oxidative stress, we measured three well-established markers of oxidative damage: DNA oxidation, protein carbonylation, and lipid peroxidation (see Figure S3). DNA oxidation was quantified using an ELISA-based assay targeting 8-hydroxy-2′-deoxyguanosine and related oxidized guanine species, following enzymatic digestion of DNA extracted from stationary-phase cultures.^43^ Protein carbonylation was measured using a colorimetric assay based on 2,4-dinitrophenylhydrazine (DNPH), which reacts with carbonyl groups on oxidatively modified proteins to form detectable hydrazone derivatives.^44^ Lipid peroxidation was assessed by quantifying malondialdehyde (MDA) levels using the thiobarbituric acid reactive substances (TBARS) assay. MDA is a stable end-product of lipid peroxidation and serves as a reliable marker of oxidative damage to membrane lipids.^45^ No significant differences in oxidative DNA damage, protein carbonylation, or lipid peroxidation were detected in any of the TCA cycle knockouts compared to the wild type (see Figure S3). Thus, although TCA cycle disruption does not measurably affect oxidative damage, the redox response revealed by our proteomics data (Figure 1) likely reshapes proteostasis. Also, the reduced persistence of TCA cycle knockouts is unlikely to result from oxidative damage under these conditions, consistent with our previous findings.^32^

### TCA cycle disruption reprograms stationary-phase energy homeostasis

Our proteomic analysis of the Δ*sdhA* mutant revealed pronounced changes in oxidative phosphorylation, proteostasis, and lipid degradation pathways, pointing to broad remodeling of stationary-phase physiology. Guided by these findings, we evaluated corresponding phenotypic parameters, including metabolic activity, intracellular ATP levels, cytoplasmic pH, cell size and complexity, and protein aggregation. We first used Redox Sensor Green (RSG) dye, which fluoresces in response to cellular reductase activity and serves as a general indicator of bacterial metabolic activity (Figure 2B). Flow cytometry analysis of late stationary-phase cells revealed that all TCA cycle knockouts exhibited lower RSG fluorescence than WT (Figure 2B), consistent with our proteomics data indicating reduced metabolic activity (Figure 1).

ATP levels were measured in the late stationary phase using a luciferase-based assay, and interestingly, all knockouts exhibited significantly higher intracellular ATP concentrations compared to WT (Figure 2C). The observed ATP elevation persists throughout the stationary phase (see Figure S4). Although intriguing, inverse metabolic relationships in genetically perturbed strains, such as those with TCA cycle deletions or ATP synthase mutants, have also been documented in previous studies.^46^^,^^47^ We infer that the increase in ATP levels arises from reduced utilization rather than enhanced production in our study, as supported by proteomics demonstrating decreased TCA cycle and ETC activity alongside a broader metabolic downshift (Figure 1). If ATP synthase and respiratory activity are reduced in the knockout strains, we would expect impaired proton export and a potential shift toward fermentative metabolism (leading to the accumulation of organic acids), both of which contribute to cytoplasmic acidification.

Therefore, we next measured intracellular pH using the pH-sensitive GFP variant pHluorin, expressed from an inducible plasmid (Figure 2D). The pHluorin reporter has a dual excitation peak at 410 and 470 nm with an emission maximum at 530 nm.^48^^,^^49^ Changes in intracellular pH alter the excitation ratio, enabling ratiometric measurement.^48^^,^^49^ All TCA cycle knockouts displayed significant cytoplasmic acidification relative to WT in late stationary phase (Figure 2D), further supporting a global metabolic downshift in these strains and corroborating the findings of our proteomics analysis (Figure 1).

### TCA cycle disruption alters stationary-phase cellular architecture

Next, we employed flow cytometry to assess apparent cell size and intracellular complexity using forward scatter (FSC) and side scatter (SSC) signals, respectively (Figures 2E and 2F). During the stationary phase, self-digestion of proteins and membrane phospholipids typically drives cell shrinkage and loss of cytoplasmic content, reducing both parameters, which are expected to reduce FSC and SSC signals due to decreased cell size, intracellular density, and structural complexity. Our previous work showed that inhibiting stationary-phase metabolism suppresses self-digestion.^32^ Consistent with this, TCA cycle knockouts generally showed increased (FSC, Figure 2E), indicating larger cell size, and elevated (SSC, Figure 2F), reflecting greater intracellular complexity and preservation of cellular content. These findings were validated by phase-contrast microscopy for selected knockout strains (Figure 2G). Visual inspection and quantitative cell perimeter (Figure 2G) analysis confirmed that knockout cells were significantly larger than WT cells in the stationary phase (Figure 2H). Notably, these effects were specific to the stationary phase, as no consistent trends were observed in FSC and SSC profiles during exponential growth (see Figure S5).

Microscopy also revealed differences in protein aggregation. WT cells frequently showed bright polar foci in phase contrast images, likely corresponding to large polar aggregates (LPAs), while these structures were less pronounced in the TCA cycle knockouts (Figure 2G). These aggregates are a hallmark of aging stationary-phase cells and have been associated with delayed regrowth and persister cell formation.^50^^,^^51^^,^^52^^,^^53^^,^^54^ To further investigate these LPAs quantitatively in stationary-phase cells, we employed a low-copy plasmid-based reporter system (pUA66-*ftsZ-gfp*), in which the *ftsZ* gene is fused to *gfp* and placed under the control of an isopropyl β-D-1-thiogalactopyranoside (IPTG)-inducible T5 promoter (Figure 2I). FtsZ is an essential cytoskeletal protein that orchestrates bacterial cell division by forming the Z-ring at the division site.^55^^,^^56^^,^^57^^,^^58^ We selected FtsZ-GFP as a reporter for two key reasons: (1) under stress conditions, FtsZ has been shown to localize to protein aggregates, making it a suitable proxy for visualizing and quantifying these structures, and (2) Z-ring formation is critical for the resumption of growth in dormant cells, directly linking FtsZ dynamics to persistence and antibiotic susceptibility.^50^^,^^51^^,^^52^^,^^53^^,^^55^^,^^56^^,^^57^^,^^58^^,^^59^^,^^60^

To visualize Z-ring formation and protein aggregation, late stationary-phase cells from WT, Δ*sdhA*, and Δ*sucC* strains harboring the FtsZ reporter plasmid were transferred to phosphate-buffered saline (PBS) agarose pads and imaged using fluorescence microscopy (Figure 2I). Based on the localization patterns of FtsZ-GFP, we observed highly heterogeneous structures, including well-formed single Z-rings, multiple Z-rings within the same cell, filamentous morphologies, FtsZ-enriched LPAs, mid-cell localization of FtsZ proteins, and in some cases, a complete absence of detectable FtsZ signal (Figure 2I). Nearly half of WT cells displayed clear FtsZ-enriched LPAs, whereas this phenotype was significantly reduced in both the Δ*sdhA* and Δ*sucC* mutants (Figure 2J). This analysis was performed on large sample sizes: 7,391 WT cells, 6,201 Δ*sdhA* cells, and 5,494 Δ*sucC* cells. In general, Z-ring-like structures were more frequently observed in the mutants compared to WT, although these were also heterogeneous, often appearing as multiple rings or as mid-cell FtsZ assemblies rather than polar foci (Figure 2K). Thus, despite the structural variability, a clear reduction in LPA formation was evident in the mutants relative to WT, indicating that loss of LPAs can be associated with reduced persistence in TCA cycle knockouts (Figures 2I–2K).

**Figure 3:**
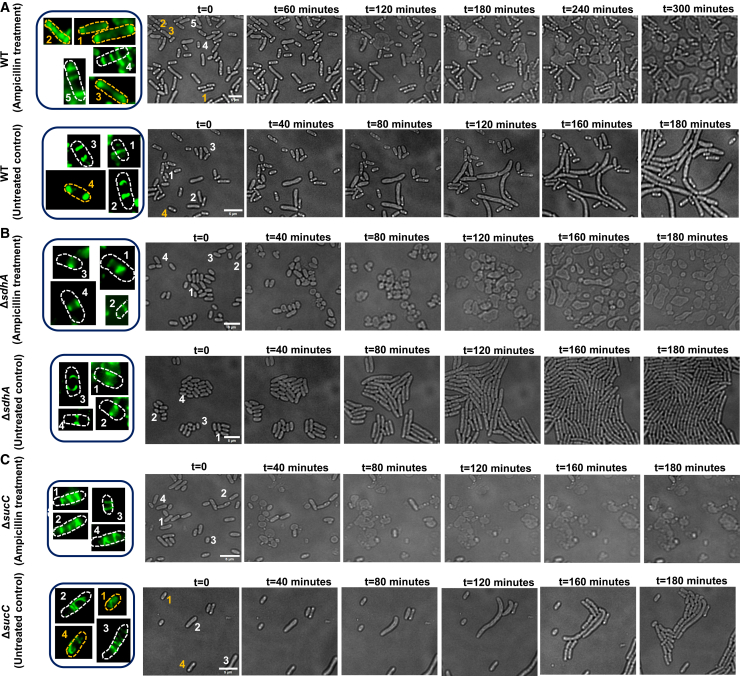
Time-lapse microscopy reveals rapid ampicillin-induced lysis of Δ*sdhA* and Δ*sucC* cells relative to WT Time-lapse microscopy of (A) WT, (B) Δ*sdhA*, and (C) Δ*sucC* strains under untreated and ampicillin-treated conditions (200 μg/mL ampicillin). Each image contains two rows: ampicillin-treated cells (top) and untreated controls (bottom). In each row, the first column shows a fluorescent image of representative cells at *t* = 0 h (FtsZ-GFP signal used to visualize Z-rings associated with cell division), with numbered cells outlined by dotted lines to indicate orientation; orange marks non-growing or unlysed cells, and white marks growing or lysed cells. The second column shows the corresponding phase-contrast image at *t* = 0 h, and subsequent columns show phase-contrast images at the indicated time points. Representative numbered cells are tracked over time to compare growth, survival, and lysis behaviors following antibiotic exposure. Fluorescent images were acquired only at *t* = 0 h to visualize Z-rings; repeated blue-laser excitation was avoided due to light sensitivity of the reporter and its potential to perturb growth and lysis dynamics. Images were collected at 37°C in an onstage incubator. Cell lysis or growth events can shift cell orientation and position during the time course. A representative image is shown; similar results were obtained across four biological replicates (*N* = 4). Scale bars, 5 μm. See also Video S1.

To further investigate the relationship between persistence and LPAs, we monitored individual cells by time-lapse microscopy during antibiotic exposure. Late stationary-phase cultures were inoculated onto LB agarose pads containing ampicillin (200 μg/mL), sealed with coverslips, and imaged in an onstage incubation chamber maintained at 37°C. Cells were monitored for up to 5 h, as longer time courses were limited by agarose pad deformation and loss of microscopy focus. Ampicillin was chosen for these experiments because it facilitates real-time visualization of cell death. Ampicillin-induced lysis was highly dynamic, with cells becoming spherical spheroplasts or protoplasts before rupturing, collapsing, and releasing their contents (Video S1). By contrast, untreated controls continued to elongate and divide normally (Figure 3). We observed that Δ*sdhA* cells, which exhibit markedly reduced LPAs, underwent nearly complete lysis within 3 h of ampicillin treatment, with lytic events detectable as early as 40 min (Figure 3B). Similarly, Δ*sucC* cells were also rapidly lysed, with only a small fraction of intact cells remaining (Figure 3C). In contrast, WT cells exhibited delayed lysis, and a substantial fraction of survivors retained LPAs and protein aggregates up to 5 h (Figure 3A). These single-cell observations provide direct mechanistic support for the elevated persister levels seen in WT relative to TCA cycle mutants, in line with our study.^27^ Although the connection between persistence and protein aggregation is well established in literature, our finding that enhanced proteostasis, driven by metabolic perturbations in TCA cycle mutants, sensitizes cells to antibiotic-induced lysis represents, to our knowledge, a novel link between energy metabolism, protein quality control, and antibiotic tolerance.

**Figure 4:**
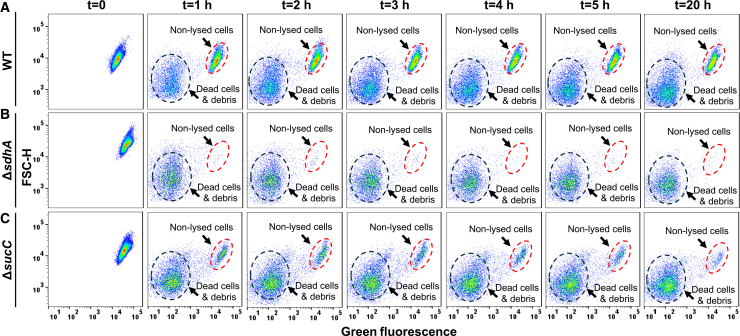
Flow cytometry analysis reveals reduced populations of intact cells in metabolic mutants Cells harboring the GFP reporter plasmid pUA66-*gfp* were cultured in the presence of inducer (1 mM IPTG). At the late stationary phase (24 h), cultures were diluted 100-fold into fresh medium containing 200 μg/mL ampicillin and incubated under the same conditions used for microscopy assays. GFP fluorescence of individual cells was monitored by flow cytometry for (A) WT, (B) Δ*sdhA*, and (C) Δ*sucC*. Upon antibiotic-induced lysis, cells lose GFP signal, whereas intact cells retain high GFP fluorescence. Flow cytometry profiles at the indicated time points are shown following FSC/SSC gating to exclude debris and non-cellular events. Intact cells were identified as GFP-positive populations retaining characteristic bacterial scatter properties, whereas lysed populations exhibited reduced FSC/SSC signals together with loss of GFP fluorescence. Similar trends were observed across four biological replicates (*N* = 4). See also Figure S6.

Because overexpression of FtsZ-GFP can potentially perturb divisome function and alter cell size (Figures 2I and 3), potentially affecting lysis dynamics, and because microscopy-based analyses sample a limited number of cells, we sought to independently validate antibiotic-induced lysis at the population level using an orthogonal approach. To this end, we employed a control reporter plasmid (pUA66-*gfp*) that uses the same vector backbone, promoter, and induction conditions as the FtsZ-GFP construct, but expresses GFP alone. We have extensively used this reporter, as well as closely related constructs, in our previous studies,^59^^,^^61^^,^^62^^,^^63^^,^^64^ persisters and viable but nonculturable (VBNC) cells, as previously validated.^61^^,^^62^ Using this assay, stationary-phase cells expressing GFP were treated with ampicillin under the same conditions used for microscopy (Figure 4). Upon lysis, cells rapidly lost GFP fluorescence, whereas intact cells retained GFP (Figure 4). Consistent with our single-cell observations, WT cultures maintained a substantial fraction of GFP-positive intact cells, even after prolonged antibiotic exposure of up to 20 h (Figures 4A and S6). In contrast, Δ*sucC* cultures showed a marked reduction in the fraction of intact GFP-positive cells, although a small subpopulation remained detectable (Figures 4C and S6). Expectedly, Δ*sdhA* cultures exhibited near-complete loss of GFP-positive cells, with intact cells becoming almost undetectable, closely mirroring the rapid and extensive lysis observed by time-lapse microscopy (Figures 4B and S6). Together, these population-level measurements corroborate our imaging data and further support the conclusion that disruption of TCA cycle-dependent metabolism profoundly sensitizes stationary-phase cells to antibiotic-induced lysis.

Altogether, the results in Figures 2, 3, and 4 demonstrate that disruption of the TCA cycle alters core aspects of stationary-phase physiology by suppressing metabolic activity, altering ATP levels, disrupting pH homeostasis, enlarging cell size, and preventing protein aggregation and mediating Z-rings. These changes underscore the TCA cycle’s role in cellular remodeling and proteostasis during stationary-phase adaptation. Notably, the differences in protein aggregation between WT and mutant strains are unlikely to result from oxidative damage (see Figure S3) but may instead reflect altered proteostasis associated with shifts in protein quality control, redox-balancing, and defense pathways in Δ*sdhA*, as highlighted by our proteomic analysis (Figure 1). Furthermore, the consistency between these metabolic and physiological observations (Figure 2) and our proteomic data (Figure 1) underlines the overall robustness and validity of our study.

### Keio knockout screening reveals key metabolic contributors to stationary-phase persistence

**Figure 5:**
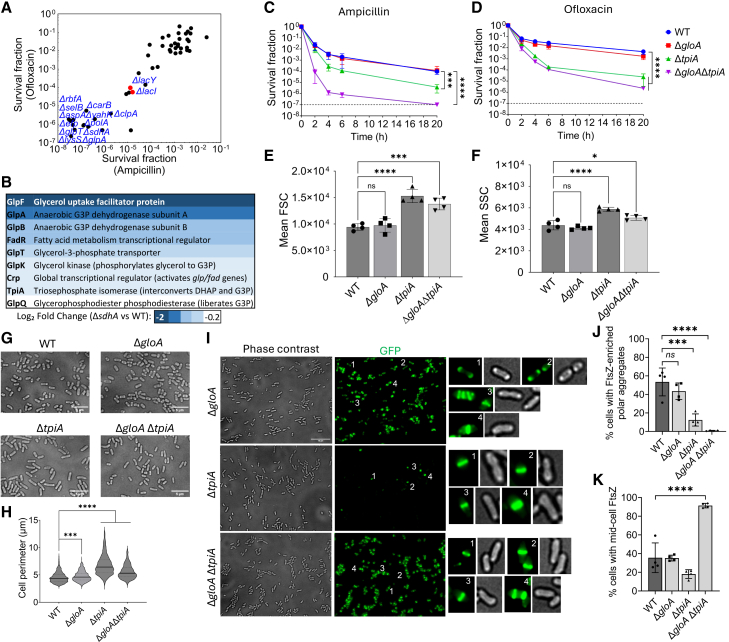
Metabolic mutants associated with Δ*sdhA* proteomic changes exhibit altered antibiotic tolerance, increased cell size, and reduced protein aggregation (A) High-throughput screening of Keio collection mutants corresponding to genes associated with significantly upregulated or downregulated proteins identified in the proteomics analysis. *lacI* and *lacY* were used as controls. Stationary-phase (24 h) cells were diluted 100-fold in fresh medium and treated with ampicillin (200 μg/mL) or ofloxacin (5 μg/mL) for 20 h. Treated cultures were washed, serially diluted, and plated to quantify colony-forming units (CFUs). Survival fraction was defined as the ratio of CFUs after treatment to CFUs measured immediately before antibiotic exposure (*N* = 1). (B) Network representation of lipid biosynthesis and degradation pathways, highlighting proteins downregulated in the Δ*sdhA* mutant compared to wild type. The color code indicates log2-transformed fold change in protein abundance (*N* = 3). (C and D) Kill curves for Δ*gloA*, Δ*tpiA*, and the double knockout Δ*gloA*Δ*tpiA* following treatment with ampicillin (200 μg/mL, C) or ofloxacin (5 μg/mL, D) (*N* = 4). (E and F) Forward scatter (E) and side scatter (F) measurements of mutant and wild-type strains by flow cytometry during late stationary phase (*N* = 4). (G) Phase-contrast micrographs of mutant and wild-type cells. A representative image is shown; similar results were obtained across four biological replicates (*N* = 4). Scale bars, 5 μm. Cell perimeters were quantified using ImageJ. (H) Quantification of cell perimeters from 1,000 individual cells per condition. (I) Microscope imaging to visualize structural organization of Z-rings and large polar aggregates in *E. coli* WT, Δ*gloA*, Δ*tpiA*, and Δ*gloA*Δ*tpiA* strains. Representative phase-contrast and corresponding fluorescence images are shown, with selected cells (numbered) enlarged and displayed side by side to highlight diverse morphologies and structural features. (*N* = 4). All biological replicates produced similar results. Scale bars, 10 μm. (J) Quantification of the percentage of cells containing large polar aggregates (LPAs) in *E. coli* WT, Δ*gloA*, Δ*tpiA* and Δ*gloA*Δ*tpiA* strains. Each dot represents the mean of a biological replicate, based on analysis of thousands of cells across multiple images (*N* = 4). (K) Quantification of the percentage of cells containing Z-rings in *E. coli* WT, Δ*gloA*, Δ*tpiA*, and Δ*gloA*Δ*tpiA* strains. Each dot represents the mean of a biological replicate, based on analysis of thousands of cells across multiple images (*N* = 4). All statistical analyses were performed using one-way ANOVA with Dunnett’s multiple comparisons test. For kill curves, ANOVA was performed on the final time points. Statistical significance is indicated as follows: ns, not significant; ∗*p* < 0.05; ∗∗∗*p* < 0.001; ∗∗∗∗*p* < 0.0001. Data represent the mean ± standard deviation. See also Figures S7, S8, S9, S10, and S11: Tables S3, S4, and S5.

To investigate the molecular basis underlying reduced persistence in TCA cycle knockouts, particularly the Δ*sdhA* strain, we performed a targeted genetic screen using the Keio *E. coli* BW25113 knockout collection (Figure 5A). Although our proteomics analysis revealed major changes in stationary-phase physiology, the specific genes driving the low-persister phenotype remained unclear. To address this, we selected 50 key genes from the Δ*sdhA* proteomic dataset (see Table S2) and assessed the persistence levels of their corresponding knockout strains. We specifically focused on the key proteins identified in the upregulated and downregulated clusters highlighted in Figures 1B and 1D, as these represent functional groups most likely to influence energy metabolism, stress adaptation, and proteostasis. For the experiments, equal numbers of stationary-phase cells were diluted in fresh media (∼100-fold) and treated for 20 h with ampicillin (200 μg/mL) or ofloxacin (5 μg/mL) at levels well above the minimum inhibitory concentrations (MICs) and commonly used to study highly tolerant persister cells. Control strains included *lacY* and *lacI* knockouts, which are not expected to influence persistence^65^ but, like all Keio strains, carry a kanamycin resistance marker. Kanamycin was added to all cultures for selection and contamination control.

Our screen identified several knockouts with reduced persister levels, most corresponding to genes downregulated in the Δ*sdhA* proteome, including SelB, RbfA, Efp, CarB, GlpT, LysS, ClpA, AspA, GlpA, and PolA (Figure 5A). This supports the hypothesis that loss of these proteins contributes to the reduced persistence observed in Δ*sdhA*. While some knockouts showed elevated persistence (see Table S2), these were generally linked to genes upregulated in Δ*sdhA*. If deletion of these genes increases persistence, their upregulation in the mutant may act to counterbalance or limit persistence. Although these findings are intriguing and will be explored in future work, our current focus is on knockouts with reduced persistence, as they are most likely to reveal how metabolic disruption suppresses persistence and to provide direct functional links to the observed proteomic changes.

Many of these genes, whose knockouts reduced persistence, align with the physiological alterations observed in the TCA cycle knockouts. For instance, SelB, RbfA, and Efp are involved in translation and ribosome function, processes tightly linked to cell growth and stress adaptation (Figure 1). The *clpA* gene encodes a key ATP-dependent protease involved in protein quality control and degradation, and its loss may contribute to the altered proteostasis and reduced protein degradation. Notably, *glpT* and *glpA* are central to glycerol metabolism, supplying intermediates to the TCA cycle as an alternative route.^37^ This pathway was found to be downregulated in the Δ*sdhA* proteome (Figure 1), indicating this route might be a critical factor of stationary-phase persistence.

### Glycerol catabolism sustains persistence in stationary-phase cells

We are particularly interested in glycerol metabolism because we hypothesize that stationary-phase cells in nutrient-depleted conditions catabolize phospholipids, generating glycerol that feeds into central metabolism to sustain proton motive force (PMF). While our initial analysis of proteomics data revealed broad metabolic changes (Figure 1), motivated by results from our Keio screen, we revisited our proteomic data with a specific focus on lipid-associated pathways (see data and code availability) and observed consistent downregulation of key proteins required for phospholipid degradation and glycerol processing in the Δ*sdhA* mutant. These include glycerol (GlpF), GlpA, GlpB, glycerol-3-phosphate transport (GlpT), GlpK, TpiA, glycerol-3-phosphate (GlpQ), and the global regulator Crp (Figure 5B). These proteins mediate periplasmic degradation of glycerophosphodiesters to GlpQ; GlpF, and periplasmic GlpT; phosphorylation and conversion into glycolytic intermediates (GlpK, TpiA); redox-linked processing (GlpA, GlpB); and transcriptional regulation under nutrient limitation (Crp),^66^ which serves as the global regulator of this alternative glycerol metabolism.^37^ Furthermore, we re-examined two metabolomics datasets generated under comparable stationary-phase culture conditions in our previous studies and identified changes consistent with phospholipid-derived glycerol metabolism (see Tables S3 and S4).^27^^,^^67^ Although those earlier analyses primarily focused on the energy metabolism of persisters rather than lipid pathways, the data clearly showed that major intracellular *E. coli* phospholipid components (which are abundant constituents of the cytoplasmic membrane) and glycerol-3-phosphate decreased at late stationary phase relative to early stationary phase,^27^ indicating active phospholipid utilization (see Table S3). In contrast, in cells treated with a PMF inhibitor, which experience collapsed PMF and reduced TCA and ETC activity similar to our metabolic mutants, the same lipids and glycerol-3-phosphate accumulated markedly,^67^ suggesting their limited utilization (see Table S4). While all of these data clearly point to the importance of phospholipid degradation (Figure 5B) and disruption of some of the key enzymes within them (e.g., GlpABC and GlpD, see Figure 8A) has also been shown by others to reduce persistence,^68^ whether glycerol-derived metabolites are directly used by stationary-phase persisters to sustain PMF and confer antibiotic tolerance remains unresolved.

To investigate this, we deleted *tpiA*, *gloA*, and both genes in *E. coli* MG1655 and performed selected key assays as described in the previous sections. We specifically focused on TpiA and GloA because they represent metabolic bottlenecks: TpiA maintains carbon flux by interconverting dihydroxyacetone phosphate and glyceraldehyde-3-phosphate, while GloA detoxifies methylglyoxal, a byproduct of elevated glycerol metabolism, feeding into an alternative TCA route (see Figure 8A). Although the Δ*tpiA* and the double mutant Δ*gloA*Δ*tpiA* exhibited slower growth compared to the wild type (see Figure S7), flow cytometry-based cell counts confirmed that the total number of cells in late stationary phase was similar across all strains (see Figure S8). Membrane integrity also remained mostly intact in these strains (see Figure S9). While these gene deletions did not drastically alter MICs of *E. coli* (see Table S5), persister levels derived from the stationary phase were significantly reduced in the Δ*tpiA* mutant, with an even more pronounced decrease observed in the double mutant (Figures 5C and 5D). Additionally, since persister cell colonies in the *tpiA* and double mutants grow slowly, assay plates were incubated longer to ensure small colonies had sufficient time to develop and become countable (see Figures S10 and S11). FSC and SSC analyses revealed that Δ*tpiA* and Δ*gloA*Δ*tpiA* cells were larger and exhibited greater intracellular complexity compared to WT cells (Figures 5E and 5F). These findings were validated by microscopy and quantitative measurements of cell dimensions (Figures 5G and 5H). Additionally, Δ*tpiA* and Δ*gloA*Δ*tpiA* mutants displayed reduced levels of LPAs (Figure 5G), suggesting improved proteostasis.

To further examine protein aggregation, we quantified FtsZ-GFP localization in Δ*gloA*, Δ*tpiA*, and Δ*gloA*Δ*tpiA* mutants (Figure 5I). Quantitative analysis included 10,622 Δ*gloA*, 6,787 Δ*tpiA*, and 6,150 Δ*gloA*Δ*tpiA* cells. As expected, Δ*gloA* resembled the WT strain, showing no significant differences in LPA or Z-ring formation compared to WT (Figures 5I and 5J). In contrast, both Δ*tpiA* and the double mutant exhibited significantly fewer LPAs (Figures 5I and 5J). Z-ring formation was markedly increased in the Δ*gloA*Δ*tpiA* mutant, but not in Δ*tpiA* alone (Figures 5I and 5K). Non-polar aggregates were rarely observed in the double mutant (Figures 5I–5K). Interestingly, the Δ*tpiA* mutant showed relatively low levels of detectable fluorescence and few Z-rings (which may warrant future independent investigation), but still displayed reduced LPAs compared to WT.

**Figure 6:**
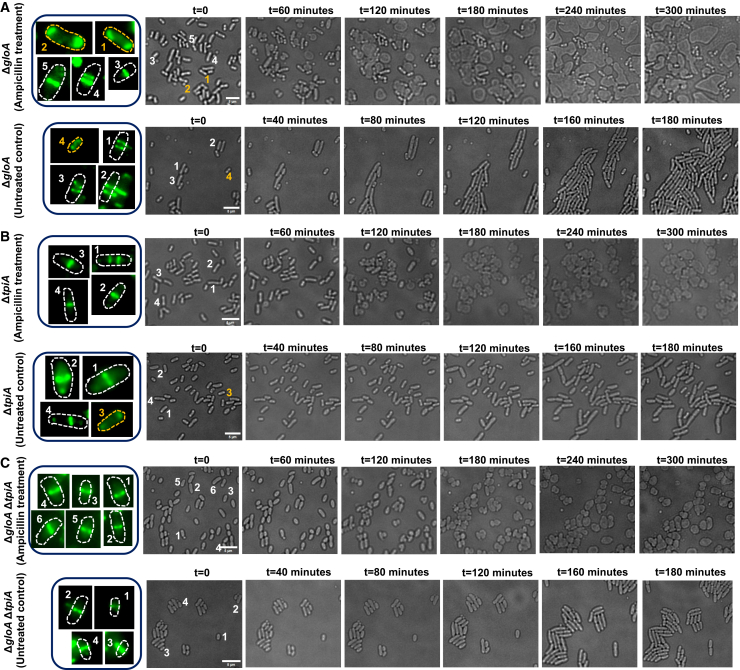
Time-lapse microscopy reveals rapid ampicillin-induced lysis of Δ*gloA*, Δ*tpiA*, and Δ*gloA*Δ*tpiA* cells Time-lapse microscopy of (A) Δ*gloA*, (B) Δ*tpiA*, and (C) Δ*gloA*Δ*tpiA* strains under untreated and ampicillin-treated conditions (200 μg/mL ampicillin). Each image contains two rows: ampicillin-treated cells (top) and untreated controls (bottom). In each row, the first column shows a fluorescent image of representative cells at *t* = 0 h (FtsZ-GFP), with numbered cells outlined by dotted lines to indicate orientation; orange marks non-growing or unlysed cells, and white marks growing or lysed cells. The second column shows the corresponding phase-contrast image at *t* = 0 h, and subsequent columns show phase-contrast images at the indicated time points. Fluorescent images were acquired only at *t* = 0 h to visualize Z-rings; repeated blue-laser excitation was avoided due to light sensitivity of the reporter and its potential to perturb growth and lysis dynamics. Images were collected at 37°C in an onstage incubator. Cell lysis or growth events can shift cell orientation and position during the time course. A representative image is shown; similar results were obtained across four biological replicates (*N* = 4). Scale bars, 5 μm.

These results (Figures 5I–5K) further reinforce the link between LPAs and persistence, suggesting that polar aggregates support stress tolerance and delayed regrowth, thereby promoting survival under antibiotic challenge. Therefore, we similarly performed time-lapse microscopy of cells exposed to ampicillin. As expected, a substantial fraction of Δ*gloA* cells remained intact after antibiotic treatment, consistent with the presence of LPAs (Figure 6A). Even after 5 h of exposure, many Δ*gloA* cells lacking Z-rings but containing LPAs persisted without lysis (Figure 6A), similar to WT (Figure 3A). On the other hand, Δ*tpiA* and Δ*gloA* Δ*tpiA* mutants were mostly lysed within 3 h, displaying the same morphological sequence observed in the Δ*sdhA* and Δ*sucC* mutants (Figures 6B and 6C).

**Figure 7:**
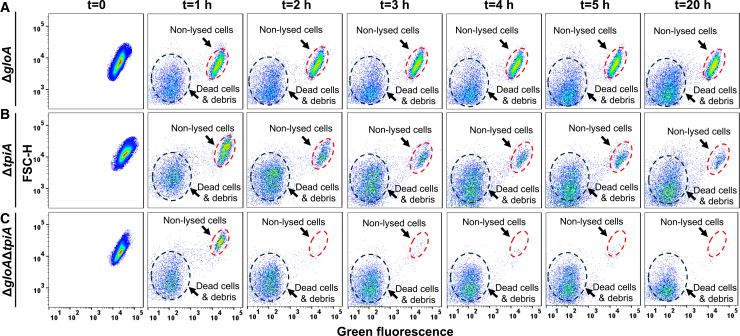
Flow cytometry analysis reveals reduced populations of intact cells in glycerol metabolism mutants Cells harboring the GFP reporter plasmid pUA66-*gfp* were cultured in the presence of inducer (1 mM IPTG). At the late stationary phase, cultures were diluted 100-fold into fresh medium containing ampicillin and incubated under the same conditions used for microscopy assays. GFP fluorescence of individual cells was monitored by flow cytometry for (A) Δ*gloA*, (B) Δ*tpiA*, and (C) Δ*gloA*Δ*tpiA*. Upon antibiotic-induced lysis, cells lose GFP signal, whereas intact cells retain high GFP fluorescence. Flow cytometry profiles at the indicated time points are shown following FSC/SSC gating to exclude debris and non-cellular events. Intact cells were identified as GFP-positive populations retaining characteristic bacterial scatter properties, whereas lysed populations exhibited reduced FSC/SSC signals together with loss of GFP fluorescence. Similar trends were observed across four biological replicates (*N* = 4). See also Figure S12.

To validate these observations at the population level and to exclude potential artifacts associated with FtsZ-GFP expression, we performed the same GFP-based lysis control assay described in the previous section using the pUA66-*gfp* reporter. Consistent with the microscopy data, Δ*gloA* cultures retained a substantial population of intact GFP-positive cells following ampicillin treatment, comparable to WT (Figures 7A and S12). In contrast, Δ*tpiA* and Δ*glo*AΔ*tpiA* cultures exhibited a pronounced loss of GFP signal compared to WT (Figures 7B, 7C, and S12), with the double mutant showing almost no detectable intact cells at the end of the treatment, indicating extensive lysis (Figures 7C and S12). These orthogonal measurements further support the conclusion that glycerol-dependent metabolic capacity and LPA formation are tightly linked to persistence and antibiotic-induced lysis outcomes.

Altogether, these observations are consistent with the phenotypes observed in TCA cycle mutants (Figures 2 and 3). The pronounced impact of *tpiA* deletion (Figures 5C–5K) reflects its central role in maintaining glycolytic flux via DHAP-G3P interconversion, creating a metabolic bottleneck. The further reduction in persistence seen in the double mutant suggests that *gloA*-mediated detoxification of methylglyoxal provides an additional survival advantage under stress.

### Glycerol catabolism sustains PMF in stationary-phase persisters

**Figure 8:**
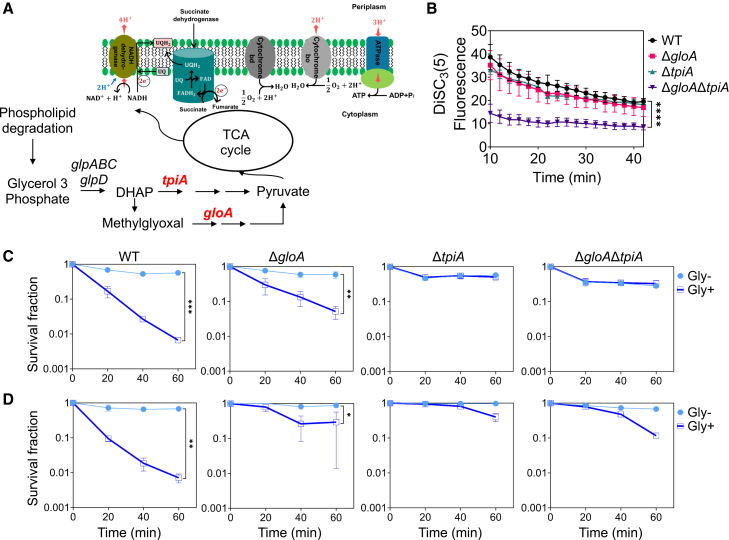
Glycerol metabolism contributes to proton motive force generation and aminoglycoside potentiation in antibiotic-tolerant cells (A) Simplified metabolic network illustrating how glycerol metabolism contributes to PMF generation. Together, TpiA and GloA form a metabolic bottleneck for glycerol-derived metabolites feeding into the TCA cycle. (B) PMF measurement using the membrane potential-sensitive dye DiSC_3_(5). Equal numbers of cells from each strain at the late stationary phase were incubated with DiSC_3_(5), and equilibrium fluorescence was recorded to assess relative membrane potential as a proxy for PMF levels. A significant difference in PMF was observed between the WT and the double-knockout strain (*N* = 4). (C and D) Aminoglycoside potentiation assay in antibiotic-tolerant persister cells. Late stationary-phase cultures were treated with 200 μg/mL ampicillin (C) or 5 μg/mL ofloxacin (D) for 20 h, then incubated with kanamycin (25 μg/mL) in M9 minimal medium supplemented with either glycerol (Gly^+^) (60 mM) or deionized water (glycerol negative, Gly^−^) as a control. Enhanced killing following glycerol supplementation reflects increased aminoglycoside potentiation through metabolic activation and PMF-dependent. Statistical significance testing (see below) was performed for the earlier time points (0, 20, and 40 min) to detect the initial aminoglycoside responses of persister cells (*N* = 4). Statistical analyses were conducted using simple linear regression models, and F-tests were employed to compare the slopes of the fitted regression lines to assess significant differences among the datasets. Statistical significance is indicated as follows: ∗*p* < 0.05; ∗∗*p* < 0.01; ∗∗∗*p* < 0.001; ∗∗∗∗*p* < 0.0001. Data represent the mean ± standard deviation. See also Figures S13, S14, S15, S16, S17, S18, and S19.

Given the importance of TCA- and ETC-mediated oxidative phosphorylation in shaping persister phenotypes observed in our results (Figures 1, 2, and 3), and the potential impact of the Δ*gloA*Δ*tpiA* mutant on PMF, a key energy parameter in persister physiology (Figure 8A), we directly measured membrane potential using 3,3′-Dipropylthiadicarbocyanine iodide [DiSC_3_(5)]. PMF is the electrochemical potential generated by a difference in proton concentration (ΔpH) and electric charge across a membrane (ΔΨ), mediated by the ETC components such as NADH dehydrogenase, succinate dehydrogenase, cytochromes, and ATP synthase, which transport protons from the TCA cycle products like NADH and flavin adenine dinucleotide (FADH_2_) across the membrane (Figure 8A), and we already shown that genetic perturbation of these key components reduces persistence under the same conditions studied here (thus, these results are not repeated here).^27^ DiSC_3_(5) is a potentiometric dye that accumulates in polarized membranes, where it self-quenches, resulting in low fluorescence.^69^^,^^70^^,^^71^ Upon membrane depolarization, the dye is released into the medium, leading to increased fluorescence (see Figure S13). Conversely, hyperpolarization enhances dye accumulation and quenching, further reducing the fluorescent signal.^69^^,^^70^^,^^71^ For these experiments, stationary-phase cells from each strain were incubated with DiSC_3_(5) under identical conditions, and fluorescence was measured after equilibrium was reached. Equilibrium fluorescence reflects the steady-state membrane potential and allows direct comparison between strains. The WT strain exhibited higher fluorescence than Δ*gloA*Δ*tpiA* mutants, indicating that the double knockout strain maintains a more hyperpolarized membrane (Figure 8B). This shift potentially reflects impaired proton export due to reduced ETC activity and increased intracellular acidification (Figure 2D). Importantly, both membrane depolarization and hyperpolarization indicate disruption of PMF homeostasis, reflecting a perturbation of the free energy defined by ΔpH and ΔΨ, which is required for ATP synthesis,^69^^,^^70^^,^^71^ and this suggests that the deletions affect membrane energetics in a physiologically substantial way.

While DiSC_3_(5) measures PMF at the population level, it does not resolve persister-specific metabolism. To overcome this, we used an AG potentiation assay, which detects PMF-dependent AG uptake triggered by specific metabolites selectively in persister cells.^24^ We previously validated this method as a functional readout of persister metabolism and used it in a high-throughput screen, identifying glycerol and glycerol-3-phosphate as among the most efficiently utilized compounds by persisters.^62^ Although the mechanism was initially unclear to us in that study, revisiting these data alongside our current proteomic and metabolic analyses (Figures 1, 2, 3, 4, 5, 6, and 7) supports the view that stationary-phase persisters retain a selective capacity for energy metabolism, with glycerol catabolism playing a central role in PMF restoration (Figure 5B; Tables S3 and S4). To further validate this hypothesis, we applied the AG potentiation assay by resuspending antibiotic-treated WT and knockout strains (Δ*tpiA*, Δ*gloA*, and Δ*gloA*Δ*tpiA*) in minimal M9 medium, with or without glycerol, supplemented with an AG (e.g., kanamycin), followed by quantification of colony-forming units (CFU) levels during the assay.^62^ If persisters actively use glycerol to restore PMF, this rapid metabolic engagement should enhance AG uptake and killing (Figures 8C and 8D). Since ampicillin persisters were around or below the limit of detection (Figure 5C), we used high cell density treatment cultures to enrich persister levels (see STAR Methods); this adjustment did not affect the observed trends (see Figure S14). We specifically focused on AG potentiation responses at early assay time points (see Figures S15 and S16), as prolonged incubation could allow persisters to resume growth and obscure their tolerant physiological state. Indeed, persisters arising from late stationary-phase cultures remain in a prolonged drug-tolerant state before growth resumes,^59^ and we have already extensively verified in our previous study that early time point measurements of AG potentiation assays provide the clearest window into initial metabolic engagement.^62^ In the presence of glycerol, both ampicillin and ofloxacin WT persisters showed enhanced AG susceptibility, whereas the Δ*tpiA* and Δ*gloA*Δ*tpiA* mutants did not, and Δ*gloA* showed an intermediate response (Figures 8C, 8D, S15, and S16). To exclude the possibility that the enhanced killing observed in the presence of glycerol reflects persister awakening or growth resumption rather than PMF restoration, we performed parallel control experiments in which antibiotic-treated persisters were incubated with or without glycerol in the absence of AGs (see Figures S17, S18, and S19). Over the duration of the AG potentiation assay, no increase in CFU or evidence of cell division was detected in any strain, indicating that glycerol alone does not trigger persister exit or growth under these conditions (see Figures S17, S18, and S19). Also, we note that in the double mutants, ofloxacin persister levels were slightly reduced during the AG assay (Figure 8D). However, this reduction was not specific to AG potentiation, as a similar decrease was observed when these cells were incubated with glycerol in the absence of AGs (see Figure S17), indicating sustained sensitivity of the double mutants under these conditions even after ending antibiotic treatment. Nevertheless, the increased susceptibility of WT persisters observed upon glycerol addition reflects metabolite-driven PMF reactivation that enables AG uptake, rather than persister wake-up or replication. The absence of both GloA and TpiA disrupts this process, establishing it as a critical metabolic bottleneck and further supporting our proposed model linking lipid-derived glycerol catabolism to persister survival.

## Discussion

Our findings reveal a mechanistic link between lipid degradation and glycerol-driven PMF generation in stationary-phase persisters. Studying the stationary phase is particularly relevant, as it reflects the nutrient-limited conditions that bacteria frequently encounter in natural environments, where they are often not actively growing but must still maintain energy homeostasis to survive stress.^17^^,^^18^^,^^19^^,^^20^ Proteomic profiling of the TCA cycle mutant, Δ*sdhA*, revealed significant downregulation of enzymes involved in lipid catabolism and glycerol metabolism, suggesting a suppression of endogenous energy-generating pathways that rely on phospholipid turnover. Pathway enrichment analysis reinforced this, highlighting lipid degradation and glycerol utilization as two of the most downregulated metabolic programs. Complementary to these observations, our Keio screen identified multiple genes upstream in lipid and glycerol metabolism whose deletion significantly reduced persistence. These findings align with existing literature; although investigations into these pathways have been sporadic, several studies report that perturbation of specific genes involved in upstream lipid and glycerol metabolism can influence persister levels and/or metabolic activity.^68^^,^^72^^,^^73^ Notably, previous AG potentiation assays demonstrated that glycerol and related metabolites are among the most effective carbon sources for sensitizing stationary-phase persisters to AGs, outperforming other common sugars like glucose, lactose, and fructose.^62^ This effect occurs not through the initiation of new metabolic programs (since phenotypic switching in late stationary phase persisters typically requires extended times) but via retained metabolic capacity that enables rapid PMF restoration upon carbon source availability.

To determine whether reduced persistence in TCA cycle mutants results from changes in oxidative stress, we directly measured DNA oxidation, protein carbonylation, and lipid peroxidation in stationary-phase cultures. Despite observing robust upregulation of oxidative stress response proteins (such as, MsrA/B, SodA, GrxA, and BtuE) in Δ*sdhA*, we found no significant differences in oxidative damage compared to the WT. These findings argue against oxidative macromolecular damage as one of the drivers of persistence in stationary phase. Our results are further supported by prior work under matched experimental conditions, which demonstrated that overexpression of catalases and superoxide dismutases did not affect persister levels, and that anaerobic respiration (absent reactive oxygen species, ROS production) still supported persistence.^32^ Together, these data indicate that while respiration can be necessary for persister formation, it does not act through ROS-induced damage. Although the elevated expression of oxidative stress defense proteins in the TCA cycle mutant seems counterintuitive, it likely reflects a compensatory redox-balancing response to altered or impaired electron flow, rather than an indication of actual oxidative injury. This defense response also contributes to protein homeostasis, potentially explaining the reduced protein aggregation observed in the mutant strains.

In our previous study, we directly tested the role of fatty acid oxidation in stationary-phase persistence by deleting key genes in the β-oxidation pathway, and these deletions had no significant impact on persister formation, indicating that long-chain fatty acid catabolism may not be required for survival under nutrient-limited, stationary-phase conditions.^32^ Our current proteomic analysis highlights the glycerol branch of lipid metabolism, with consistent downregulation of enzymes involved in this pathway in Δ*sdhA*. This metabolic preference makes physiological sense as fatty acid oxidation is highly reducing and energy-rich, but it is also oxygen-intensive and potentially ROS-generating,^74^ conditions that may be unfavorable during stationary phase. In contrast, glycerol may offer a lower-intensity, redox-balanced substrate that supports basal respiration and PMF maintenance. Glycerol can be rapidly converted into central metabolic intermediates,^37^ making it a fast and efficient route to sustain energy homeostasis during prolonged stress.

The marked reduction or absence of LPAs and the increased Z-ring formation in TCA cycle mutants compared to WT *E. coli* during stationary phase suggests improved proteostasis and altered stress physiology resulting from metabolic rewiring, and importantly, provides a mechanistic explanation for their reduced persistence. LPAs are known to accumulate in aging, nutrient-depleted cells and have been previously linked to delayed regrowth and increased antibiotic tolerance,^50^^,^^51^^,^^52^^,^^53^ making their reduction in TCA mutants particularly notable. Several mechanisms are likely to contribute to this phenotype. First, the lower metabolic activity observed in knockouts (evidenced by reduced RSG fluorescence and downregulation of energy-generating pathways) may reduce proteotoxic stress, limiting the formation of misfolded or damaged proteins.^75^ Second, proteomic data revealed upregulation of proteases and redox-balancing enzymes, which enhanced protein quality control and may prevent the accumulation of irreversible aggregates.^76^ Third, elevated ATP levels in most knockouts may support energy-dependent proteostasis pathways, including the activity of chaperones and proteases that maintain a soluble cytoplasmic protein pool.^54^^,^^77^ Finally, downregulation of ribosomal proteins and translation factors suggests reduced translational burden, thereby lowering the production of aggregation-prone nascent peptides.^78^

The decrease in LPA formation also helps explain the unexpected increase in SSC signals in TCA knockouts. In WT stationary-phase cells, LPAs localize misfolded proteins into polar foci, potentially removing them from the bulk cytoplasm and reducing cytoplasmic complexity. Additionally, WT cells often undergo significant intracellular degradation under nutrient stress, leading to cellular shrinkage and reduced internal content,^32^^,^^79^ both factors that contribute to lower SSC and FSC. This shrinkage is also expected to alter cell membrane architecture, reducing its surface area and potentially releasing phospholipids that can be recycled as internal carbon sources. In contrast, the elevated SSC and FSC in TCA knockouts likely reflects a more intact, granular cytoplasmic architecture (rather than reflecting protein aggregation), and larger cell size. Together, these observations suggest that TCA cycle disruption preserves cellular health during stationary phase and reduces the proteotoxic features associated with persistence. By limiting LPA formation and maintaining protein homeostasis, these cells may avoid the physiological states that promote drug tolerance,^54^ offering mechanistic insight into their reduced persister phenotype.

AG potentiation assays provide a sensitive readout of persister cell metabolism because AGs require active PMF for uptake and lethality.^24^^,^^62^ When desired carbon sources are added, persister cells rapidly restores PMF and becomes susceptible to AG killing.^62^ This immediate response (before any significant outgrowth) reflects intrinsic metabolic capability rather than regrowth-driven susceptibility. Importantly, persisters derived from late stationary-phase cultures exhibit extended drug-tolerant states before resuming growth.^59^ Therefore, focusing on early time points allows us to capture the initial metabolic engagement that defines the persister state, avoiding confounding effects of recovery or division. Our data show that glycerol rapidly sensitizes WT persisters to AG, suggesting that these cells either retain latent energy-generating capacity or rapidly activate key metabolic processes such as PMF generation, which itself may require transcriptional and translational activity. This observation supports the central hypothesis of our study that persister cells are not metabolically inert, but instead depend on selective, tightly regulated energy pathways for survival. The ability of persisters to quickly utilize glycerol and restore PMF implies an organized and responsive metabolic infrastructure.

Interestingly, our results also showed a slight reduction in ofloxacin-treated persister levels observed in the double mutants; however, this is not attributable to AG potentiation per se. This behavior is not unexpected given the increased antibiotic sensitivity of these mutants. Ofloxacin is known to inflict DNA damage in persister cells as well as in antibiotic-sensitive populations, such that persister survival depends on effective damage repair during the recovery phase.^23^ The increased vulnerability of the double mutants may compromise this repair capacity, leading to reduced apparent persister survival independent of AG uptake.

The deletion of key TCA cycle genes actually enhanced survival against AGs in the exponential phase.^7^^,^^47^ Additionally, metabolomic profiling revealed only minor changes in overall metabolic activity and AG uptake in TCA cycle mutants compared to WT during the exponential phase.^47^ The disparity in findings underscores that the metabolic requirements for persistence vary not only by cell type and genetic background but also by the environmental and experimental conditions under which persistence is measured. This discrepancy may be explained by the significant downregulation of ribosomal proteins observed in TCA cycle knockout strains during exponential growth, leading to reduced translation and diminished AG efficacy.^47^ Similarly, a recent study showed that ETC (*nuo*) mutants displayed increased AG tolerance during exponential growth, a phenotype linked to intracellular acidification and reduced protein synthesis.^49^ In contrast, although our metabolic mutants also exhibited increased cytoplasmic acidification during late stationary phase, these strains showed markedly reduced survival to ampicillin and ofloxacin. These differences suggest that intracellular acidification alone is unlikely to be the primary determinant of persistence in our conditions, and instead may reflect a secondary physiological consequence of disrupted energy metabolism. Together, these observations highlight the adaptive flexibility and redundancy of bacterial survival strategies, which are tightly shaped by physiological state and environmental context.

### Limitations of the study

A limitation of this study is that our proteomic analysis was restricted to the Δ*sdhA* mutant as a representative model of TCA cycle disruption rather than being performed across all persistence-deficient TCA cycle mutants. However, the major physiological and persistence phenotypes identified through this systems-level analysis were subsequently validated across multiple independent TCA cycle and glycerol metabolism mutants using genetic, metabolic, imaging, and functional assays. In addition, although our findings support a role for phospholipid-derived glycerol catabolism in persistence, direct measurement of metabolic flux within purified persister populations remains technically challenging because persisters represent a rare and heterogeneous subpopulation that cannot be easily isolated with complete purity. Nevertheless, the convergence of proteomic, metabolomic, genetic, physiological, and single-cell analyses provides multiple independent lines of evidence supporting the proposed model. Future studies integrating metabolic flux analysis with emerging approaches for persister enrichment and characterization will further refine our understanding of how glycerol metabolism supports antibiotic tolerance.

## Resource availability

### Lead contact

Requests for further information and resources should be directed to and will be fulfilled by the lead contact, Mehmet A. Orman (maorman@wisc.edu).

### Materials availability

This study did not generate new unique reagents. All mutant strains created in this study are available from the lead contact without restriction.

### Data and code availability

• All original proteomics data have been deposited at the PRIDE archive via ProteomeXchange and are publicly available as of the date of publication. The corresponding dataset accession number is listed in the key resources table.
• This paper does not report original code.
• Any additional information required to reanalyze the data reported in this paper is available from the lead contact upon request.

## Acknowledgments

The authors would like to thank the members of Orman Lab for their help. This study was supported by NSF CAREER 2044375, NSF 2326142, and 10.13039/100000002NIH/10.13039/100000060NIAID R01-AI143643. Proteomics experiments were conducted at UT Health’s Clinical and Translational Proteomics Service Center, with the associated service fees.

## Author contributions

Conceptualization, methodology, formal analysis, writing – original draft, writing – review, and editing, H.G.N., S.G.M, and M.A.O.; investigation, H.G.N. and S.G.M.

## Declaration of interests

The authors declare no competing interests.

## STAR★Methods

### Key resources table

**Table:** 

REAGENT or RESOURCE	SOURCE	IDENTIFIER
**Bacterial and virus strains**		

*Escherichia coli* K-12 MG1655	A gift from Dr. Mark P. Brynildsen at Princeton University (Orman et al.^65^)	N/A
*Escherichia coli* K-12 BW25113	Keio Collection, Horizon Discovery, Lafayette, CO	Cat#OEC4988
*Escherichia coli* K-12 Knockout strains	See Table S6	N/A

**Chemicals, peptides, and recombinant proteins**		

Isopropyl β-D-1-thiogalactopyranoside (IPTG)	Sigma-Aldrich (St. Louis, MO)	Cat#I6758-1G
Ampicillin	Fisher Scientific (Atlanta, GA)	Cat#AAJ6097706
Ofloxacin	Fisher Scientific (Atlanta, GA)	Cat#AAJ6208003
Polymyxin B	Fisher Scientific (Atlanta, GA)	Cat#21-850-029
Glycerol	Fisher Scientific (Atlanta, GA)	Cat#AAA16205AP
Kanamycin	Fisher Scientific (Atlanta, GA)	Cat#11815024

**Critical commercial assays**		

BacTiter-Glo™ Microbial Cell Viability Assay	Promega, Wisconsin, USA	Cat#G8230
DNA/RNA Oxidative Damage (High Sensitivity) ELISA Kit	Cayman Chemical, Ann Arbor, MI, USA	Cat#589320
Protein Carbonyl Colorimetric Assay Kit	Cayman Chemical, Ann Arbor, MI, USA	Cat#10005020
OxiSelect™ TBARS Assay Kit	Cell Biolabs, Inc., San Diego, CA, USA	Cat#STA-330

**Deposited data**		

Raw and processed mass spectrometry proteomics data	PRIDE/ProteomeXchange	Pride Archive: PXD079272

**Oligonucleotides**		

Primers	See Table S7	https://www.idtdna.com/

**Recombinant DNA**		

Plasmid pUA66-*ftsZ-gfp*	Our previous study (Mohiuddin et al.^59^)	N/A
Plasmid pGFPR01	A gift from Dr. Joan Slonczewski at Kenyon College (Martinez et al.^48^)	N/A

**Software and algorithms**		

GraphPad Prism V10	GraphPad Software, Boston, MA, USA	https://www.graphpad.com/
FlowJo V10	Waters Bioscience, Ashland, OR, USA	https://www.flowjo.com/
SkanIt Software V5.0	Thermo Fisher, Waltham, MA, USA	https://www.thermofisher.com/
STRING tool V12.0	Szklarczyk et al.^35^	https://string-db.org/
Evos FL Auto 2 fluorescence microscope	Thermo Fisher, Waltham, MA, USA	Cat#AMAFD2000
Proteome Discoverer V1.4	Thermo Fisher, Waltham, MA, USA	https://www.thermofisher.com/
Orbitrap Fusion Tribrid mass spectrometer	Thermo Fisher, Waltham, MA, USA	https://www.thermofisher.com/
Varioskan LUX Multimode Microplate Reader	Thermo Fisher, Waltham, MA, USA	Cat# VLBL00D0
Flow cytometer NovoCyte 3000RYB	ACEA Biosciences Inc., San Diego, CA, USA	Cat#45-1-1612-2663-7
ImageJ 1.54 g	Wayne Rasband and contributors National Institutes of Health, USA	https://imagej.net/ij/

### Experimental model and study participant details

*Escherichia coli* K-12 MG1655 wild type (WT) was gift from Dr. Mark P. Brynildsen at Princeton University; plasmid pGFPR01 (the pHluorin reporter) was a gift from Dr. Joan Slonczewski at Kenyon College^48^; and plasmid pUA66-*ftsZ-gfp* was obtained from our previous study.^59^ Mutant strains of *E. coli* K-12 MG1655 were generated in our previous studies,^27^ and mutants of *E. coli* K-12 BW25113 were obtained from the Keio Collection (Catalog #OEC4988, Horizon Discovery, Lafayette, CO). The *E. coli* K-12 MG1655 Δ*gloA*, Δ*tpiA*, and Δ*gloA*Δ*tpiA* knockout strains were constructed using the Datsenko–Wanner method.^80^ When necessary, the antibiotic resistance cassette was removed via FLP-mediated excision to generate markerless mutants, enabling the generation of double knockout strains.^80^ All strains and primers used to generate and verify the strains in this study are listed in Tables S6 and S7. *E. coli* MG1655 strains carrying the low-copy plasmid pUA66-*ftsZ-gfp* were induced with Isopropyl β-D-1-thiogalactopyranoside (IPTG) under the control of a synthetic T5 promoter.^32^ All chemicals were purchased from Fisher Scientific (Atlanta, GA), VWR International (Pittsburgh, PA), or Sigma-Aldrich (St. Louis, MO). Bacterial strains were cultured in liquid Luria–Bertani (LB) medium. LB agar was used for enumerating colony-forming units (CFU). LB broth was prepared by dissolving 5 g of yeast extract, 10 g of tryptone, and 10 g of sodium chloride in 1 L of deionized (DI) water, followed by autoclaving. LB agar was prepared by dissolving 40 g of premixed LB agar in 1 L of DI water. The M9 salt solution was prepared following previously described protocols.^62^ Phosphate-buffered saline (PBS, 1×) was used to wash cells free of antibiotics. Kanamycin (50 μg/mL) was added to overnight and main cultures to maintain plasmid selection. Fluorescent protein expression was induced with 1 mM IPTG. Ampicillin (200 μg/mL) and ofloxacin (5 μg/mL) were used as bactericidal antibiotics in persister assays. The minimum inhibitory concentrations (MICs) of antibiotics for WT and mutant strains were determined using Liofilchem MTS (MIC Test Strips) (Fisher Scientific, Atlanta, GA). Since persisters are known to survive high antibiotic concentrations, we used doses significantly above the MICs (Table S5), consistent with the literature. Ampicillin was dissolved in DI water, whereas ofloxacin was dissolved in 0.01 N sodium hydroxide prepared in DI water. Polymyxin B (32 μg/mL), glycerol (600 mM), kanamycin (25 μg/mL), and IPTG (1 M) stock solutions were prepared in DI water. All chemical solutions were sterilized using 0.2 μm VWR syringe filters. Overnight cultures (ONs) were prepared in 14 mL Falcon tubes containing 2 mL of LB medium, inoculated from glycerol stocks (25%) stored at −80°C, and incubated at 37°C with shaking at 250 rpm for 24 h. Main cultures were initiated by diluting ONs 1:1000 in 2 mL of fresh LB medium. Under these conditions, cultures typically reached mid-exponential phase after 3 h and late stationary phase after 24 h. Stationary-phase cells from the main cultures were used for persister, metabolic, and morphological characterization assays, as these cultures reach the stationary phase more uniformly. This consistency results from the controlled initial inoculum size during the main culture setup. In contrast, direct use of ON cultures can lead to variability due to uncontrolled initial cell numbers, often resulting in inconsistent growth phase progression.^32^

### Method details

#### Cell growth and persister assays

Growth curves of the WT and mutant strains were generated by measuring the optical density at 600 nm (OD_600_) of the main cultures for the indicated time points. At each time point, samples from the main culture and fresh media (used as a blank) were added to a 96-well plate and read at 600 nm using a Varioskan LUX Multimode Microplate Reader (Thermo Fisher, Waltham, MA, USA). Data were collected using SkanIt Software v5.0.

For persister assays, overnight cultures of *E. coli* K-12 (MG1655 WT and BW25113) and their knockout strains were diluted 1:1000 into fresh LB medium. Cultures were prepared in 14 mL Falcon tubes and incubated at 37°C with shaking at 250 rpm. Upon reaching the late stationary phase, the cultures were further diluted 1:100 into fresh LB medium supplemented with antibiotics (ampicillin at 200 μg/mL and ofloxacin at 5 μg/mL). These cultures were then incubated again at 37°C with shaking for 20 h. After antibiotic treatment, 1 mL from each culture was transferred to a microcentrifuge tube and centrifuged at 13,300 rpm (17,000 × g). The supernatant (950 μL) was discarded, and the cell pellets were washed twice with 1× PBS to reduce residual antibiotic concentrations below the MIC. The washed cells were then resuspended in 100 μL of 1× PBS. A 10 μL aliquot of this suspension was serially diluted in a 96-well round-bottom plate. Subsequently, 10 μL from each dilution was spotted onto LB agar plates. In addition, 90 μL from the most concentrated well was plated to ensure detection of viable cells down to a limit of 1 CFU/mL. Plates were incubated at 37°C for up to 72 h to allow colony formation. Colony counts were initiated after 16 h of incubation for fast-growing strains. For strains with slower colony development, 48 h of incubation was typically sufficient. To generate kill curves, 100 μL samples were collected at the indicated time points during treatment, washed, and plated as described above to quantify changes in CFU over time. For BW25113 mutants, LB medium contained 50 μg/mL kanamycin to prevent potential contamination due to the large number of samples, although each strain was grown in a separate test tube.

#### Proteomics analysis

Proteins were isolated from the *sdhA* knockout and WT strains of *E. coli* K-12 MG1655 at the late stationary phase (24-h main culture) and sent to UT Health’s Clinical and Translational Proteomics Service Center (Houston, TX) for mass spectrometry analysis. Because the same experimental workflow was used and explained in our previous study, we provide a concise summary here, with additional details available in the referenced publication.^27^ Cell lysates were prepared using NEBExpress *E. coli* Lysis Reagent (NEB, Catalog# P8116S) and total protein concentrations were quantified using the bicinchoninic acid (BCA) assay (Thermo Fisher Scientific, Catalog# 23225). Equal amounts of protein from each sample were subjected to acetone precipitation, followed by denaturation/reduction (6 M urea, DTT), alkylation (iodoacetamide), and overnight tryptic digestion. Peptides were desalted using reverse-phase cartridges and dried by vacuum centrifugation prior to LC-MS/MS analysis. Approximately 1 μg of tryptic digest was analyzed using an Orbitrap Fusion Tribrid mass spectrometer (Thermo Fisher Scientific) coupled to a Dionex UltiMate 3000 RSLCnano system. Peptides were separated on a C18 analytical column and eluted using a gradient of acetonitrile containing 0.1% formic acid at a flow rate of 350 nL/min. Data were acquired in data-dependent acquisition mode, with MS1 spectra collected in the Orbitrap (resolution 120,000; m/z 350–1500) followed by MS2 acquisition using higher-energy collisional dissociation (HCD). Dynamic exclusion was applied for 35 s. Raw data were processed using Proteome Discoverer (v1.4, Thermo Fisher Scientific) and searched against the *E. coli* Swiss-Prot database using the Sequest HT search engine. Spectra were filtered using a target false discovery rate (FDR) of 1%, allowing up to two missed tryptic cleavages. Carbamidomethylation of cysteine was included as a fixed modification, whereas methionine oxidation and asparagine deamidation were included as variable modifications.

Data processing followed the analysis steps outlined by Aguilan et al.^34^ *p*-value calculation was performed using a parametric *t* test.^34^ The selection of the appropriate *t* test type was guided by an F-test, which evaluated whether the replicates for each protein exhibited homoscedasticity (equal variances) or heteroscedasticity (unequal variances).^34^ The STRING tool (version 12.0) was used to identify significant protein–protein interaction networks among the input proteins.^35^ For protein network and pathway enrichment analysis, we used protein identifiers (accession numbers) for proteins that were upregulated or downregulated by at least 2-fold. STRING-based protein network analysis identified biological pathways that were significantly overrepresented, based on the statistical background of the entire genome and the set of proteins altered in our proteomics analysis. Network edges were defined based on “evidence,” prioritizing different types of interaction evidence to support automated pathway enrichment analysis. Pathway enrichment analysis was performed using multiple functional classification frameworks, including Gene Ontology annotations, KEGG pathways, and UniProt keywords, as previously described.^35^ Enrichment was quantified using a “strength score,” calculated as Log_10_(observed/expected), where higher values indicate stronger enrichment of a given biological pathway. This score reflects the ratio between (i) the number of proteins annotated with a specific term in the observed network and (ii) the expected number in a random network of the same size. Statistical significance was assessed using the False Discovery Rate, with *p*-values corrected for multiple comparisons within each category using the Benjamini–Hochberg procedure.^35^

For the volcano plot, we included proteins that were significantly upregulated or downregulated, based on our data analysis following the workflow described by Aguilan et al.^34^ The threshold for upregulation was set at a fold-change of 2, as indicated by vertical dotted lines on the x axis. Statistical significance was defined as a *p*-value ≤0.05, marked by a horizontal dotted line on the y axis. Color coding was used to illustrate changes in protein levels in the knockout strain compared to wild type: red dots represent upregulated proteins, with corresponding genes listed in the red-outlined box; blue dots represent downregulated proteins, with corresponding genes listed in the blue-outlined box (Figure 1A). Genes in both boxes are ordered by the magnitude of fold-change, from highest upregulation to highest downregulation (Figure 1A). Gray dots represent proteins that do not meet the significance threshold.

#### Flow cytometry analysis for FSC and SSC measurements

Main cultures of *E. coli* K-12 MG1655 WT and mutant strains were prepared by diluting 24-h overnight cultures 1:1000 into 2 mL of LB medium, followed by incubation at 37°C with shaking at 250 rpm until reaching the stationary phase (24 h). Late stationary-phase cells were then diluted 1:100 in 1 mL of 1× PBS. Prior to flow cytometry analysis, the samples were gently vortexed. Cells were analyzed with a flow cytometer (NovoCyte 3000RYB, Serial #45-1-1612-2663-7, ACEA Biosciences Inc., San Diego, CA) using forward scatter (FSC) and side scatter (SSC) parameters. Samples were run at a flow rate of 14 μL/min, with a sample stream (core) diameter of 7.7 μm. The instrument maintained a constant sheath flow rate of 6.5 mL/min. The core diameter was calculated based on the ratio of the sample flow rate to the sheath flow rate. These conditions were selected to optimize data resolution for measuring *E. coli* cell size. A solvent-only control (1× PBS or assay buffer without cells) was acquired under identical instrument settings to determine the baseline electronic and optical background. Events falling within this background distribution, which were concentrated near the origin of the FSC versus SSC plot and represented instrument noise or particulate contaminants, were excluded from subsequent analysis. The main bacterial population was identified on FSC versus SSC density plots using untreated WT cells, which formed a reproducible, high-density cluster distinct from background events. A polygon gate encompassing this population was applied to exclude low-scatter debris and anomalous high-scatter aggregates. Because stationary-phase cultures contain heterogeneous cell morphologies, gates were intentionally set to include the full WT bacterial population while excluding events outside the established scatter distribution.

For fluorescence-based assays, fluorescence gates were established only after FSC/SSC-based exclusion of background and debris. Assay-specific negative and positive controls were used to define fluorescence thresholds, and these thresholds were subsequently applied consistently across all strains and experimental conditions. Additional assay-specific gating details are provided in the corresponding sections below.

#### Propidium iodide (PI) measurement

Main cultures of *E. coli* K-12 MG1655 WT and mutant strains were prepared by diluting 24-h overnight cultures 1:1000 into 2 mL of LB medium. Cultures were incubated at 37°C with shaking at 250 rpm and allowed to reach the stationary phase (24 h). PI dye (Catalog #P1304MP, Thermo Fisher, Waltham, MA) was used to assess membrane integrity. Late stationary-phase cells were diluted 1:100 in 1 mL of 0.85% NaCl buffer. To each sample, 1 μL of 20 mM PI dye was added in flow cytometry tubes. After brief vortexing to ensure uniform mixing, samples were incubated at 37°C in the dark for 10 min. Prior to fluorescence analysis, sequential gating was performed to exclude background noise and non-cellular events. A solvent-only control (0.85% NaCl buffer without cells) was first analyzed under identical instrument settings to define background signal and exclude events clustered near the origin of FSC versus SSC plots. Next, untreated live cells were used to define the intact bacterial population based on FSC and SSC characteristics and to establish the PI-negative gate. Ethanol-killed cells (70% ethanol for 30 min), which exhibited membrane permeabilization and strong PI staining, served as a positive control to define the PI-positive population and fluorescence threshold for membrane-compromised cells. Events outside the FSC/SSC distribution of intact bacterial cells were excluded as debris prior to fluorescence quantification. Following staining, samples were analyzed by flow cytometry. Cells were excited at 561 nm, and red fluorescence was detected using a 615/20 nm bandpass filter.

#### Redox measurement

Main cultures of *E. coli* K-12 MG1655 WT and mutant strains were prepared by diluting 24-h overnight cultures 1:1000 into 2 mL of LB medium, followed by incubation at 37°C with shaking at 250 rpm for 24 h to reach the stationary phase. To measure bacterial metabolic activity, Redox Sensor Green (RSG) dye (Catalog #B34954, Thermo Fisher Scientific, Waltham, MA) was used. Late stationary-phase cells were diluted 1:100 in 1 mL of 0.85% NaCl buffer, and 1 μL of RSG dye was added to each flow cytometry tube. After brief vortexing to ensure uniform mixing, samples were incubated at 37°C in the dark for 10 min. Samples were then analyzed by flow cytometry. Cells were excited at 488 nm, and green fluorescence was detected using a 530/30 nm bandpass filter. For redox measurements, assay-specific gating was applied following initial exclusion of background events. Buffer-only samples (0.85% NaCl without cells) were first acquired to identify baseline instrument noise and establish exclusion boundaries for non-cellular signals. The primary bacterial population was then identified using FSC and SSC distributions from untreated WT cells, allowing exclusion of low-scatter debris and irregular events. To validate the dynamic response of the RSG dye and establish reduced metabolic activity controls, cells were pretreated with 20 μM carbonyl cyanide *m*-chlorophenyl hydrazone (CCCP) for 5 min prior to staining, resulting in markedly reduced fluorescence signals relative to untreated cells.^27^ These CCCP-treated populations were used as reference controls for defining low-redox fluorescence distributions, and identical gating parameters were subsequently applied across all strains and experimental conditions.

#### Single-cell analysis of ampicillin-induced lysis

Overnight cultures of *E. coli* MG1655 WT, Δ*sdhA*, Δ*sucC*, Δ*gloA*, Δ*tpiA*, and Δ*gloA* Δ*tpiA* strains harboring the pUA66-*gfp* plasmid were diluted 1,000-fold into 2 mL of fresh LB medium in 14-mL Falcon tubes and grown at 37 °C with shaking for 24 h. At late stationary phase (t = 24 h), cultures were diluted 100-fold into 2 mL of fresh LB medium and immediately treated with ampicillin (200 μg/mL) for up to 20 h. At designated time points (t = 0, 1, 2, 3, 4, 5, and 20 h), treated cells were collected, diluted in 1× PBS, and analyzed by flow cytometry with the same parameters described above. To ensure plasmid maintenance and GFP expression throughout the experiment, cultures were continuously supplemented with kanamycin (50 μg/mL) and IPTG (1 mM), respectively. GFP-positive control samples (cells carrying the pUA66-*gfp* reporter and induced with IPTG) and GFP-negative control samples (cells lacking the reporter plasmid or not induced) were used to define fluorescence thresholds. PBS-only samples were first acquired to identify baseline electronic and optical background and exclude non-cellular events. Untreated GFP-expressing cells were then used to establish the FSC/SSC profile of intact bacterial cells and define the fluorescence distribution of viable GFP-positive populations. During antibiotic treatment, lysed cells were identified as events exhibiting both reduced FSC/SSC signals, consistent with membrane disruption and cellular fragmentation, and loss of GFP fluorescence relative to intact cells. Events with scatter characteristics outside the intact bacterial population and lacking GFP signal were classified as debris and excluded from intact-cell quantification. Gating boundaries established using these control populations were maintained across all strains and time points to ensure consistency in longitudinal comparisons. This gating strategy to distinguish intact, lysed, and debris populations has been validated and described in detail in our previous studies.^61^^,^^62^ and was applied consistently across all experiments.

#### Microscope imaging for cell size measurement

Main cultures of *E. coli* K-12 MG1655 WT and mutant strains were prepared by diluting 24-h overnight cultures 1:1000 into 2 mL of LB medium, followed by incubation at 37°C with shaking at 250 rpm until reaching the stationary phase (24 h). Late stationary-phase cells were then diluted 1:2 with 1× PBS, and 100 μL of the diluted suspension was transferred onto PBS agar pads. The agar pads were prepared by dissolving 1% agarose in 1× PBS, followed by microwave sterilization. The PBS agarose solution was poured over a glass slide (25 × 75 mm), following the method described previously.^59^ A glass coverslip (25 × 75 × 0.17 mm) was placed on top of the agar pad containing the cells. Phase-contrast images were acquired using a fluorescence microscope (Evos FL Auto2, Catalog #AMAFD2000, Thermo Fisher Scientific, Waltham, MA) equipped with a 100× oil immersion objective (Olympus, Catalog #AMEP4733; working distance: 0.3 mm) to assess cellular morphology. ImageJ was used to quantify the cell perimeter in 1,000 cells. To perform this analysis, we first enhanced the contrast of the microscopy images, subtracted the background, set a threshold to remove additional background noise, converted the images to binary masks, and outlined the cell membranes. Finally, we applied filtering conditions to exclude noise and analyze the cells more accurately. Scale bars in the raw images were used as references for cell length measurements.

#### LPA and Z-ring measurements

The method used to image Z-rings in this study was adapted from a previously published approach.^59^ Overnight culture of *E. coli* MG1655 WT, Δ*sdhA*, Δ*sucC*, Δ*gloA*, Δ*tpiA*, Δ*gloA* Δ*tpiA* cells harboring the pUA66-*ftsZ-gfp* plasmid were diluted 1:1000-fold in 2-mL LB broth in 14-mL test tubes and cultured for 24 h with shaking. Kanamycin (50 μg/mL) and IPTG (1 mM) were added in the cultures to maintain the plasmid and to induce *ftsZ-gfp* expression, respectively. At t = 24 h, cells were diluted 1:10-fold in PBS (1×). The diluted cells (300 μL) were then transferred to PBS agarose pads, which were dried next to a Bunsen burner flame for ∼20 min. The agarose pads were prepared by dissolving agarose (1%) in PBS followed by microwave sterilization. The PBS agarose medium was then poured over a glass slide (25 × 75 mm), each side of which was enclosed by stacked slides^59^ to make the pad smooth and sufficiently thick. Once the agar pads containing cells were dried, glass coverslips (25 × 75 × 0.1 mm) were placed on the top of the cells. Both phase-contrast and fluorescence (GFP) images were obtained using a fluorescence microscope (Evos FL Auto 2; Catalog # AMAFD2000; Thermo Fisher Scientific) with a 100× (oil) objective (Olympus, Catalog # AMEP4733; working distance, 0.3 mm) to determine cellular morphology and assess expression of the FtsZ-GFP fusion protein. An Evos GFP light cube (Catalog # AMEP4651) was used to acquire GFP images with 470/22-nm excitation and 510/42-nm emission wavelengths. Cell morphology, polar aggregates, and FtsZ ring structures in microscope images were analyzed with ImageJ software. To capture a heterogeneous cell population in each replicate, at least 8 different locations in each agarose pad, leading to a total number of cells ranging from 1000 to 2500 (*x*4 biological replicates), were selected and monitored with the use of the Evos FL Auto 2 imaging system. The brightness of the phase-contrast images and the color of the fluorescent images were adjusted with “brightness/contrast,” “color balance,” and “sharpen” options. The polar aggregates and Z-rings within a bacterium was enumerated manually using the fluorescent images.

#### Time-lapse microscope imaging

Main cultures of *E. coli* K-12 MG1655 WT and mutant strains were prepared by diluting 24 h overnight cultures 1:1000 into 2 mL of LB medium and incubating at 37 °C with shaking (250 rpm) until stationary phase (24 h). Late stationary-phase cells were then diluted 1:5 in fresh LB medium, and 100 μL of the suspension was transferred onto LB agarose pads. Agarose pads were prepared by dissolving 1% agarose in LB medium, sterilizing the solution by microwaving, supplementing with ampicillin, and pouring it onto glass slides (25 × 75 mm). To maintain the pUA66*-ftsZ-gfp* plasmid and induce expression, 50 μg/mL kanamycin and 1 mM IPTG were added, respectively. For ampicillin treatment, a final concentration of 200 μg/mL was used in agar pads, consistent with the persister assay. Untreated pads served as controls. Time-lapse imaging was performed using an on-stage incubator to maintain 37 °C, air, and humidity. Phase-contrast and fluorescence (GFP) images were captured with an Evos FL Auto 2 fluorescence microscope equipped with a 100× oil immersion objective (specifics provided above). Cellular morphology and FtsZ-GFP expression were analyzed. GFP fluorescence was detected using an Evos GFP light cube with 470/22-nm excitation and 510/42-nm emission filters.

#### ATP measurement

Main cultures of *E. coli* K-12 MG1655 WT and mutant strains were prepared by diluting 24-h overnight cultures 1:1000 into 2 mL of LB medium in 14 mL Falcon tubes, followed by incubation at 37°C with shaking at 250 rpm for 24 h to reach the stationary phase. To determine cell numbers, 10 μL of the main culture at the stationary phase was analyzed by flow cytometry, as described above. Simultaneously, 100 μL of the main culture was mixed with 100 μL of ATP detection reagent from the BacTiter-Glo Microbial Cell Viability Assay (Catalog #G8230, Promega, Wisconsin, USA) in a black, flat-bottom 96-well plate. The mixture was incubated for 5 min at 37°C on the same shaker. Luminescence was measured using the Varioskan LUX Multimode Microplate Reader (Thermo Fisher, Waltham, MA, USA). Data were collected using SkanIt Software v5.0, and ATP levels were calculated based on a standard curve generated from known ATP concentrations and normalized to cell numbers.

#### pH measurement

A standard curve for intracellular pH as a function of fluorescence ratio (410/470) in *E. coli* K-12 MG1655 was generated using the previously described steps.^48^^,^^49^ Cells carrying the pGFPR01 plasmid encoding pHluorin were induced with 0.2% L-arabinose in the main culture to express the pH-sensitive fluorescent protein. The plasmid was maintained with 100 μg/mL ampicillin. To create pH buffers necessary for the standard curve, 2-(N-morpholino) ethanesulfonic acid (MES) and 3-(N-morpholino) propanesulfonic acid (MOPS) solutions were prepared at 50 mM concentrations, adjusted to pH values ranging from 5.0 to 10.0 in 0.5-unit increments. For the standard curve, first, transmembrane pH collapsed by adding 40 mM potassium benzoate and 40 mM methylamine hydrochloride to the stationary phase cultures, equalizing the difference between the external and internal pH. Then, culture samples were collected, washed to remove the chemicals, and normalized to an OD_600_ of 1.0 in 0.5 mL of each pH buffer. A 150 μL aliquot from each suspension was transferred into a 96-well plate, with LB used as the blank. The fluorescence ratio (410/470) was measured using a plate reader, and the generated standard curve was used to obtain a Boltzmann sigmoid best-fit equation^49^:

(Equation 1) $$H=6.64-0.6484\times \ln\left(\frac{1.79-0.1575}{fluorescence\;ratio-0.1572}-1\right)$$

For pH measurements in experimental samples, main cultures of *E. coli* K-12 MG1655 WT and mutant strains carrying the pGFPR01 plasmid (maintained with 100 μg/mL ampicillin and induced with 0.2% L-arabinose) were prepared by diluting 24-h overnight cultures 1:1000 into 2 mL of LB medium. Cultures were incubated in 14 mL Falcon tubes at 37°C with shaking at 250 rpm and grown to the stationary phase (24 h). Then, OD_600_ of the cultures was normalized to 1.0 in fresh LB, and 150 μL from these normalized cultures was transferred to a 96-well plate (LB as blank), and the fluorescence ratio was measured with a plate reader. This ratio was then used to determine pH based on the standard curve equation above.

#### DNA damage assay

Main cultures of *E. coli* K-12 MG1655 WT and mutant strains were prepared by diluting 24-h overnight cultures 1:1000 into 2 mL of LB medium, followed by incubation at 37°C with shaking at 250 rpm for 24 h to reach the stationary phase. Cell densities were normalized based on the OD_600_ of WT and mutant strains at 2.5, in 2 mL samples, which were then used for total DNA extraction. DNA extraction and quantification were performed using the DNeasy Blood & Tissue Kit (Catalog #69504, Qiagen, Germantown, MD, USA) according to the manufacturer’s instructions. DNA at a concentration of 25 ng/μL in a total volume of 45 μL was used for the DNA damage assay. To the extracted DNA, 5 μL of 10× Nuclease P1 Reaction Buffer (Catalog #M0660, New England BioLabs Inc., Ipswich, MA, USA) and 0.1 μL of Nuclease P1 were added. The pH of the solution was adjusted using 1 M Tris buffer, followed by the addition of 1 unit of alkaline phosphatase (*E. coli* C75) (Catalog #2120 A, Takara Bio Inc., Kusatsu, Shiga, Japan). The mixture was incubated at 37°C for 30 min, then the reaction was inactivated by heating at 75°C for 10 min. DNA damage levels were measured using the DNA/RNA Oxidative Damage (High Sensitivity) ELISA Kit (Catalog #589320, Cayman Chemical, Ann Arbor, MI, USA) according to the manufacturer’s protocol. This assay measures DNA/RNA damage caused by oxidative processes. Specifically, it detects oxidatively damaged guanine species, including 8-hydroxyguanosine, 8-hydroxy-2′-deoxyguanosine, and 8-hydroxyguanosine. The assay is an ELISA that utilizes AChE (8-OH-dG–acetylcholinesterase conjugate) technology. Equal amounts of extracted DNA from each sample are digested, and the lysates are incubated with AChE to compete for binding to antibodies specific to DNA/RNA oxidative damage. These antibody-bound complexes then attach to goat polyclonal anti-mouse IgG coated on the bottom of the 96-well plate provided by the vendor. After washing away unbound substances, the bound complexes are quantified using a reagent supplied by the vendor, which catalyzes an enzymatic reaction producing a distinct yellow color measured at 412 nm. The intensity of this color is proportional to the amount of AChE complexed with the antibodies and inversely proportional to the level of oxidative damage in the DNA/RNA samples. A standard curve for the DNA/RNA oxidative damage ELISA was generated using the assay’s provided standard solution.

#### Protein damage assay

Main cultures of *E. coli* K-12 MG1655 WT and mutant strains were prepared by diluting 24-h overnight cultures 1:1000 into 2 mL of LB medium, followed by incubation at 37°C with shaking at 250 rpm for 24 h to reach the stationary phase. Cell densities were normalized based on an OD_600_ of 2.5 for both WT and mutant strains, and 2 mL of these cells were washed twice with cold 1× PBS while kept on ice throughout the process. Centrifugation was performed at 4°C, 13,000 rpm for 3 min. The resulting pellets were lysed in 300 μL of NEBExpress *E. coli* Lysis Reagent (Catalog #P8116S, New England BioLabs Inc., Ipswich, MA, USA) at room temperature for 30 min. The lysate was then centrifuged at 16,600 × g for 10 min, and 250 μL of the supernatant was collected for the assay. Total protein concentration was determined using the Bicinchoninic Acid (BCA) assay (Catalog #23225, Thermo Fisher Scientific, Waltham, MA). Twenty-five μL of each cell lysate sample and DI water as a blank were loaded into a 96-well plate. Then, 200 μL of BCA working solution (prepared at a 50:1 ratio of Reagent A to B) was added to each well and mixed thoroughly on a plate shaker for 30 s. The plate was incubated at 37°C for 30 min and then cooled for 5 min at room temperature, protected from light. Absorbance was measured at 562 nm using a plate reader. Protein concentrations were calculated using a standard curve generated from BCA standards. Protein carbonyl levels in control and mutant strains were measured using the Protein Carbonyl Colorimetric Assay Kit (Catalog #10005020, Cayman Chemical, Ann Arbor, MI, USA) according to the manufacturer’s instructions and the detailed analysis protocol. This assay measures protein oxidation by quantifying carbonyl protein content, a common marker of oxidative damage. Carbonyl groups form when iron and copper cations bind to proteins and are subsequently attacked by reactive oxygen species, which oxidize the side-chain amine groups of certain amino acids into carbonyls. The lysed samples are then incubated with 2,4-dinitrophenylhydrazine (DNPH). DNPH reacts with the carbonyl groups to form Schiff bases, producing hydrazones that can be analyzed spectrophotometrically at 360 to 385 nm.

#### Lipid damage assay

Main cultures of *E. coli* K-12 MG1655 WT and mutant strains were prepared by diluting 24-h overnight cultures 1:1000 into 2 mL of LB medium, followed by incubation at 37°C with shaking at 250 rpm for 24 h to reach the stationary phase. Cell densities were normalized based on an OD_600_ of 2.5 for both WT and mutant strains. 1 mL of late stationary phase cells was washed twice with cold 1× PBS while kept on ice throughout the process. Centrifugation was performed at 4°C, 13,000 rpm for 3 min. After the final centrifugation, all the supernatant was removed except for 100 μL, which was used to resuspend the cell pellets. Cell density was measured by flow cytometry using 10 μL of the adjusted culture diluted in 990 μL of 1× PBS. The remaining cell culture was treated with a 100-fold excess of butylated hydroxytoluene solution to prevent further oxidation and then lysed using the SDS cell lysis buffer provided in the OxiSelect TBARS Assay Kit (Catalog #STA-330, Cell Biolabs, Inc., San Diego, CA, USA). The mixture was incubated at room temperature for 5 min, followed by the addition of 250 μL of thiobarbituric acid reagent. The mixture was then incubated for 60 min at 95°C and subsequently cooled in an ice bath for 5 min. After centrifugation at 13,000 rpm for 15 min, the supernatant was collected for further analysis. 200 μL of the supernatant were added to a 96-well plate and read at 532 nm using a Varioskan LUX Multimode Microplate Reader (Thermo Fisher, Waltham, MA, USA). Data were collected using SkanIt Software V 5.0. A standard curve for malondialdehyde (MDA) was generated using the assay’s provided standard solution. This assay measures lipid peroxidation by detecting its end-products, such as MDA, which is stable and a widely accepted marker of oxidative stress. The lysed samples are incubated with thiobarbituric acid reagent, where MDA forms a complex with thiobarbituric acid reactive substances (TBARS). This complex can be measured calorimetrically at 532 nm or fluorometrically with excitation at 540 nm and emission at 590 nm.

#### DiSC_3_(5) assay

The DiSC_3_(5) assay was performed as previously described.^69^^,^^70^^,^^71^ Main cultures of *E. coli* K-12 MG1655 WT and mutant strains were prepared by diluting 24-h overnight cultures 1:1000 into 2 mL of LB medium, followed by incubation at 37°C with shaking at 250 rpm until reaching stationary phase at 24 h. Late stationary-phase cells were collected, normalized to an OD_600_ of 0.1, and washed twice with buffer containing 5 mM HEPES (N-[2-Hydroxyethyl]piperazine-N-[2-ethanesulfonic] hemisodium salt) and 20 mM glucose. The washed cells were stained with 1 μM DiSC_3_(5) diluted 1:1000 and incubated in the dark. Fluorescence levels were measured using a plate reader at 620 nm excitation and 670 nm emission wavelengths every 2 min for up to 42 min. After 20 min, when fluorescence levels reached equilibrium, control samples were treated with polymyxin B (32 μg/mL), a membrane disruptor that binds lipopolysaccharide and induces conformational changes in the membrane, and fluorescence was remeasured for another 20 min. Polymyxin B treatment served to verify the functionality of the dye.

#### Aminoglycoside potentiation assay

The AG potentiation assay was performed as previously described.^62^ This established assay has been extensively validated as an experimental framework for assessing persister metabolism and aminoglycoside potentiation, with appropriate controls previously performed under identical conditions and shown to provide a robust proxy for metabolic activity in persister cells. M9 minimal medium was intentionally used to allow glycerol to serve as the sole carbon source and avoid confounding effects from alternative nutrients present in rich media. For this assay, the main culture was diluted 10-fold into 25 mL of fresh media in 250 mL baffled flasks and treated with ampicillin (200 μg/mL) and ofloxacin (5 μg/mL) for 20 h. Persisters were collected from the 25 mL culture, washed twice with M9 salt medium, suspended in 1 mL (cells concentrated), and divided into equal cell numbers into two test tubes containing either DI water (control) or 60 mM glycerol. Both sets were subsequently treated with kanamycin (25 μg/mL). Every 20 min, 100 μL samples were taken, washed twice with 1× PBS, serially diluted in a 96-well plate, and 10 μL from each dilution was plated onto LB agar plates. Colony-forming units were quantified after incubation at 37°C until visible colonies appeared, for up to 72 h.

### Quantification and statistical analysis

Each experiment included at least three biological replicates unless otherwise stated. Data points in the figures represent the mean ± standard deviation. For microscopy analyses, representative images from both phase-contrast and fluorescence microscopy are shown. Statistical analyses were performed using one-way ANOVA with Dunnett’s multiple comparisons test where applicable. For time-dependent responses, simple linear regression models were used, and F-tests were applied to compare the slopes of the regression lines. Thresholds for significance were set as follows: ∗*p* < 0.05, ∗∗*p* < 0.01, ∗∗∗*p* < 0.001, ∗∗∗∗*p* < 0.0001. GraphPad Prism V10 and FlowJo V10 were used to generate figures and perform statistical analyses.

## Supplemental information

Document S1. Figures S1–S19 and Tables S2–S7

Table S1. Functions of proteins are found to be upregulated or downregulated based on proteomics analysis, related to Figure 1

Video S1. Time-lapse microscopy of *E. coli* cells during ampicillin treatment, related to Figure 3 Late stationary-phase cells were immobilized on LB agarose pads containing ampicillin (200 μg/mL) and imaged at 37°C in an onstage incubator. Lysis can be a rapid and dynamic process, with cells collapsing into spherical spheroplasts or protoplasts before rupturing and releasing their intracellular contents (indicated by arrows). We note that ampicillin induced the same lysis dynamics in all *E. coli* strains tested.
