# Supplementary material for: Glycerol-driven energy and proteostasis underpin antibiotic tolerance in Escherichia coli
Source: iScience. 2026 Jul 22;29(8):116896. doi: 10.1016/j.isci.2026.116896 (PMC13427511; doi:10.1016/j.isci.2026.116896)
Supplement: Document S1. Figures S1–S19 and Tables S2–S7 [file mmc1.pdf]

iScience, Volume *29*

Supplemental information

Glycerol-driven energy and proteostasis underpin antibiotic tolerance in *Escherichia coli*

Han G. Ngo, Sayed Golam Mohiuddin, and Mehmet A. Orman

Supplementary Information

Supplementary Figures

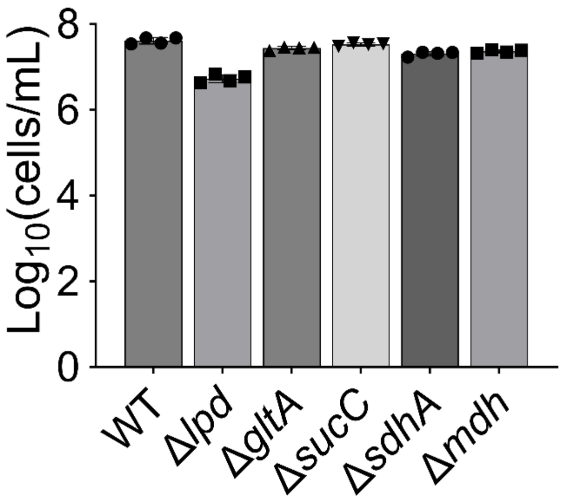

Figure S1. Flow cytometry-based cell quantification, related to Figure 2. *Escherichia coli* wild-type (WT) and mutant strains (Δ*lpd*, Δ*gltA*, Δ*sucC*, Δ*sdhA*, and Δ*mdh*) at late stationary phase (24 h) were diluted in 1× PBS and analyzed by flow cytometry to quantify cell counts. The number of biological replicates, N=4. Data represent the mean ± standard deviation.

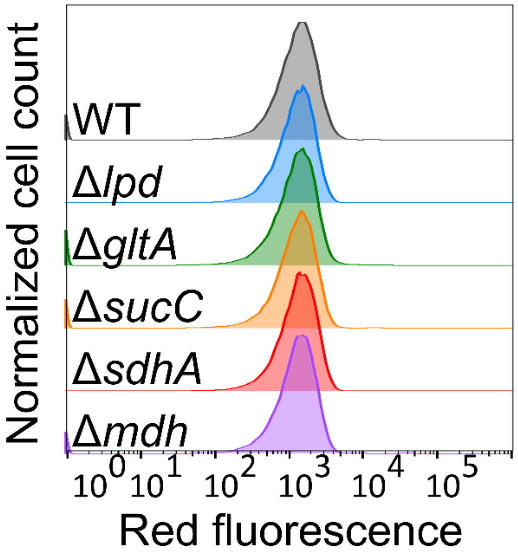

Figure S2. Propidium iodide staining of cells at late stationary phase, related to Figure 2. *E. coli* WT and mutant strains (Δ*lpd*, Δ*gltA*, Δ*sucC*, Δ*sdhA*, and Δ*mdh*) at late stationary phase (24 h) were collected, diluted in sterile 0.85% NaCl buffer solution, and then stained with 20 μM propidium iodide dye. Stained cells were analyzed by flow cytometry. A minimum of four biological replicates were conducted, with each displaying consistent trends. The flow diagram shown is representative of these results.

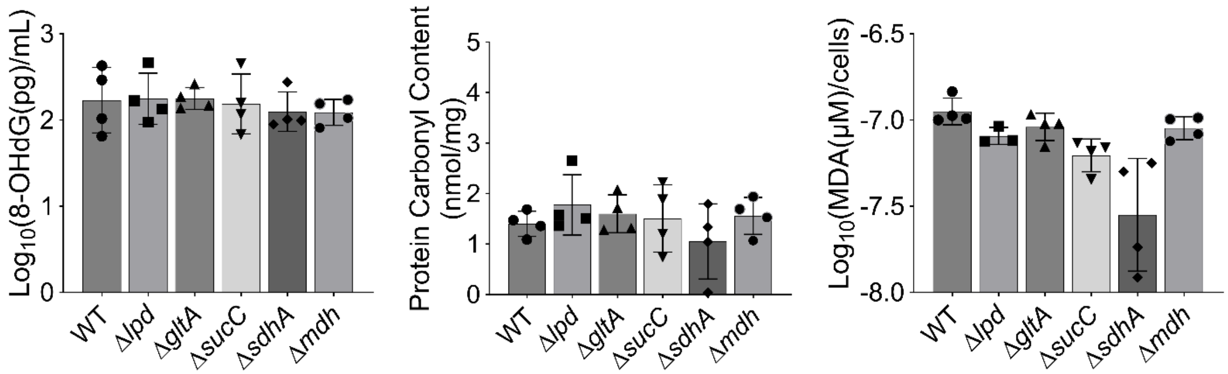

Figure S3. Assessment of oxidative stress using three biomarkers: DNA oxidation, protein carbonylation, and lipid peroxidation, related to Figure 1 and 2. Cells from the indicated strains at late stationary phase (24 h) were collected to assess DNA oxidation, protein carbonylation, and lipid peroxidation. These parameters were measured using commercial kits following the manufacturer's protocols (see **Materials and Methods**). For each condition and strain, identical cell densities were used. Protein carbonyl content and lipid damage (MDA) levels were further normalized to total protein concentration and cell number, respectively. DNA damage levels (8-OHdG) are reported as absolute concentrations, as the same amount of DNA was used for all conditions and strains. N=4. Data represent the mean ± standard deviation.

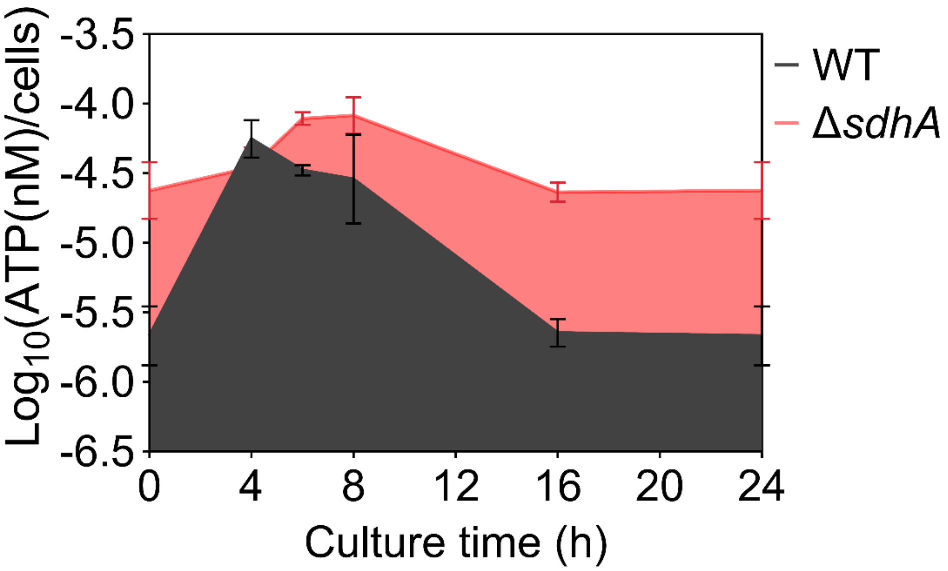

Figure S4. Quantification of intracellular ATP levels in the cells, related to Figure 2. Overnight stationary phase cultures of *E. coli* WT and Δ*sdhA* strains were diluted 1:1000-fold into fresh medium and incubated for 24 hours. At specified time points, cells were collected, and intracellular ATP levels were measured using the BacTiter-Glo™ Microbial Cell Viability Assay Kit, following the manufacturer's instructions. To normalize ATP concentrations per cell, cell counts were simultaneously determined using flow cytometry. N=4. Data represent the mean ± standard deviation.

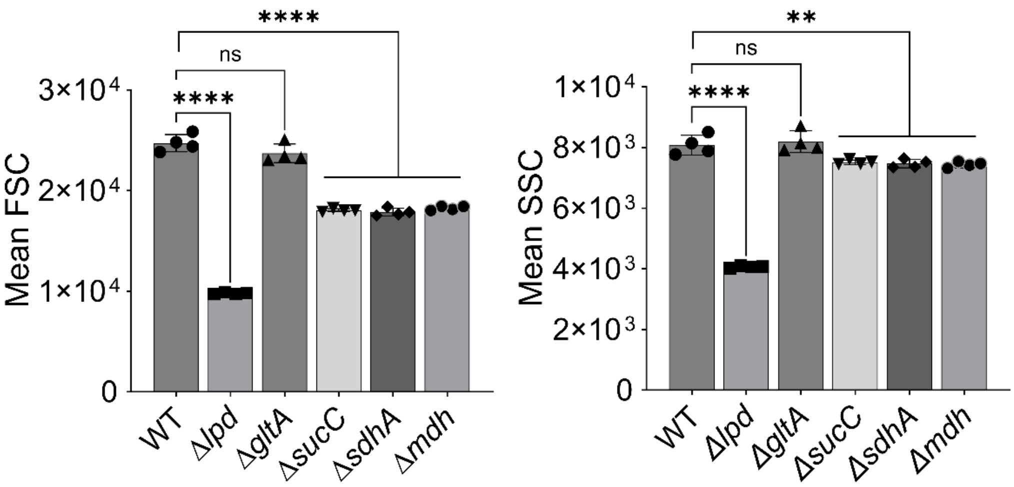

Figure S5. Morphological characterization of TCA cycle knockout strains during the exponential phase, related to Figure 2. Overnight cultures of *E. coli* WT and mutant strains (Δ*lpd*, Δ*gltA*, Δ*sucC*, Δ*sdhA*, and Δ*mdh*) were diluted 1:1000-fold in fresh LB media and grown to exponential phase (t=3 h). Then, cells were collected and diluted in 1× PBS and analyzed by flow cytometry to measure forward scatter (FSC) and side scatter (SSC) at the single-cell level. N=4. Statistical analysis was performed between the WT and single mutants using one-way ANOVA with Dunnett's post-test. *P<0.05, **P < 0.01, ***P < 0.001, and ****P < 0.0001. Data represent the mean ± standard deviation.

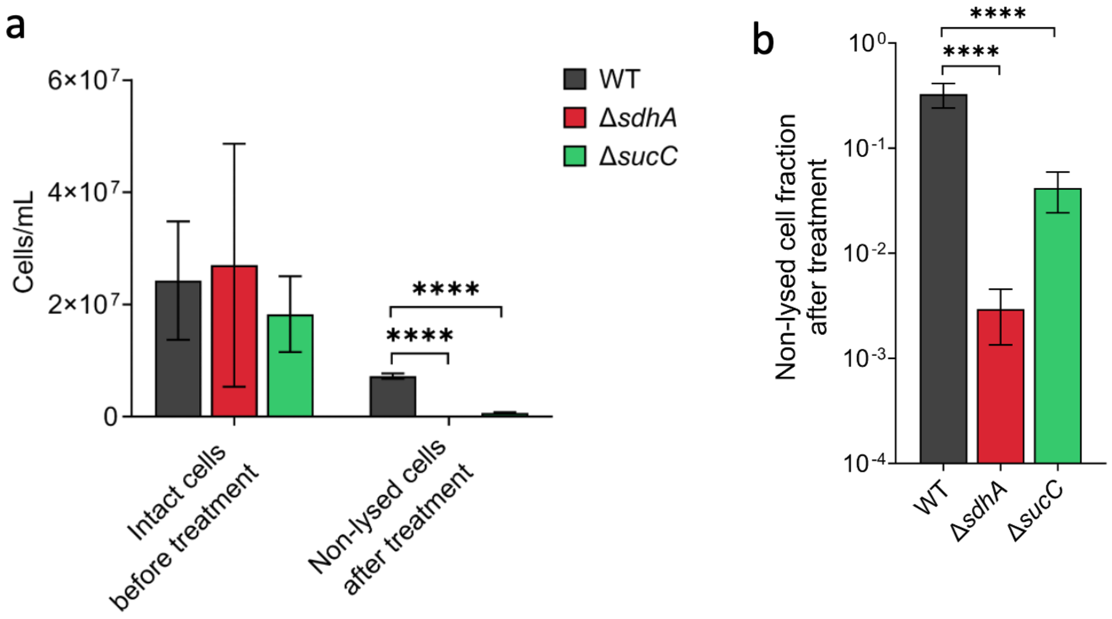

Figure S6. Flow cytometry quantification of intact cells after ampicillin treatment, related to Figure 4. (a) Intact cell (GFP-positive) numbers were measured by flow cytometry before and after 20 h of ampicillin treatment in WT, Δ*sdhA*, and Δ*sucC* strains. (b) The intact cell fraction (non-lysed cell fraction) was determined as the ratio of intact cells after treatment to the initial cell number. The graph in panel (b) is displayed on a log scale. N = 4. Statistical analysis was performed using two-way ANOVA with Dunnett's multiple comparisons test; ****$P < 0.0001$. Data represent mean ± standard deviation from four independent biological replicates.

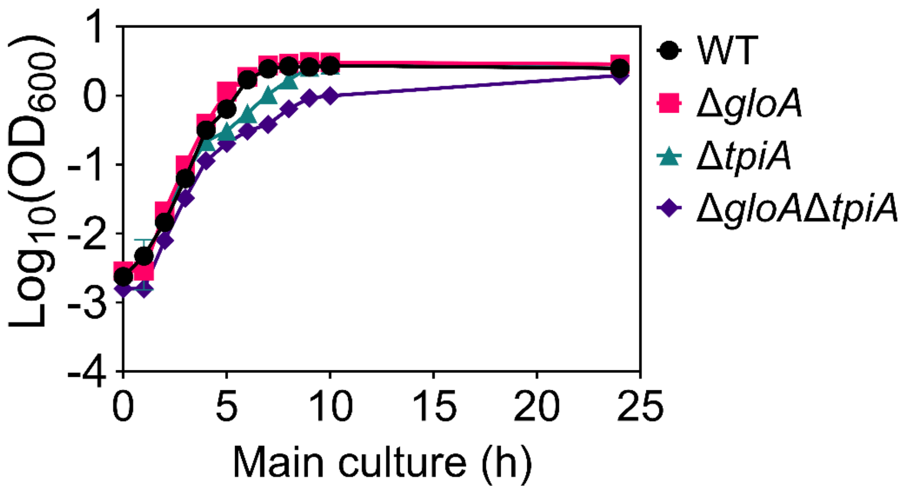

Figure S7. Growth curves of WT, Δ*gloA*, Δ*tpiA,* and Δ*gloA* Δ*tpiA* strains, related to Figure 5. Overnight cultures of *E. coli* WT and mutant strains (Δ*gloA*, Δ*tpiA*, and the double mutant Δ*gloA* Δ*tpiA*) were diluted 1:1000-fold into fresh LB medium and incubated for 24 hours at 37 °C with shaking. At designated time points, cells were collected, and optical density at 600 nm (OD_{600}) was measured using a plate reader. N=4. Data represent the mean ± standard deviation.

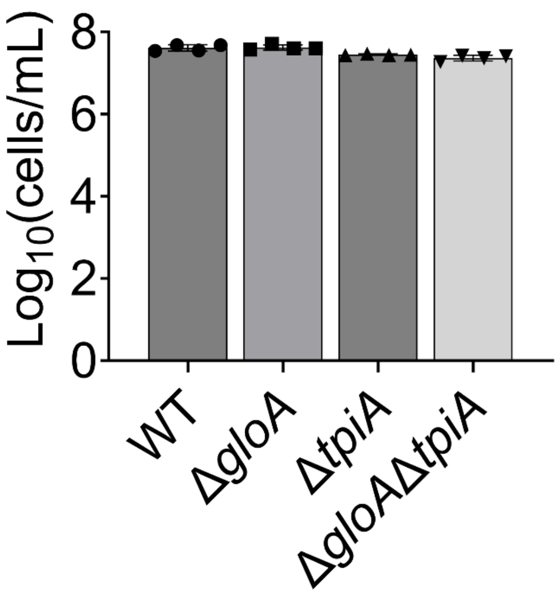

Figure S8. Flow cytometry-based cell quantification, related to Figure 5. *E. coli* WT and mutant strains (Δ*gloA*, Δ*tpiA*, and the double mutant Δ*gloA* Δ*tpiA*) at late stationary phase (24 h) were diluted in 1× PBS and analyzed by flow cytometry to quantify cell counts. N=4. Data represent the mean ± standard deviation.

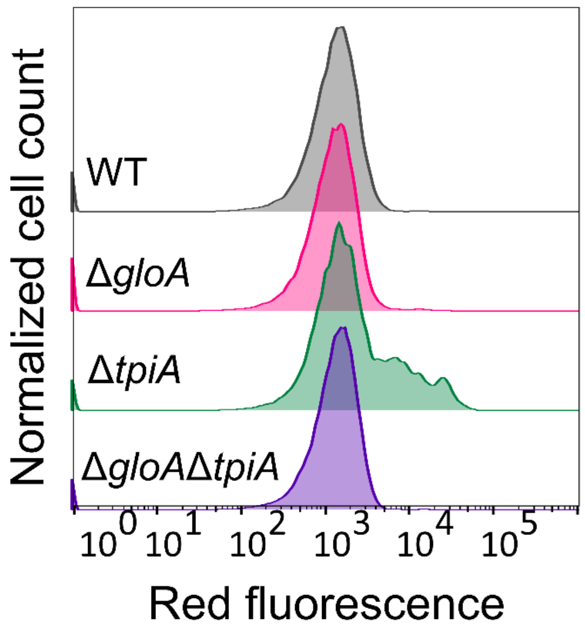

Figure S9. Propidium iodide staining of cells at late stationary phase, related to Figure 5. *E. coli* WT and mutant strains (Δ*gloA*, Δ*tpiA*, and the double mutant Δ*gloA* Δ*tpiA*) at late stationary phase (24 h) were collected, diluted in sterile 0.85% NaCl buffer solution, and then stained with 20 μM propidium iodide dye. Stained cells were analyzed by flow cytometry. A minimum of four biological replicates were conducted, with each displaying consistent trends. The flow diagram shown is representative of these results.

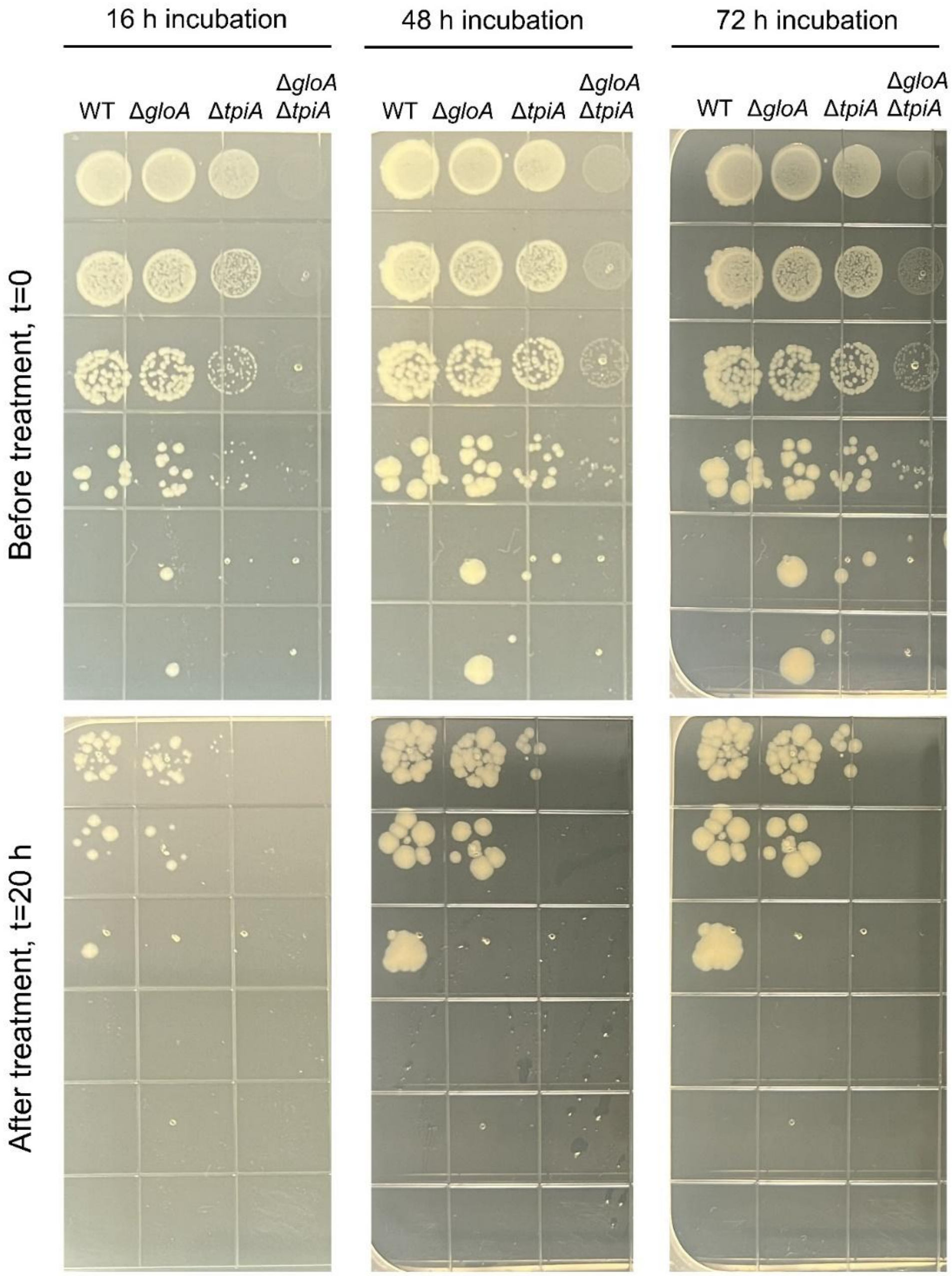

Figure S10. Colony formation of WT and mutant strains visualized on agar plates, related to Figure 5. Late-stationary-phase (24 h) cultures of *E. coli* WT, Δ*gloA*, Δ*tpiA*, and the double mutant Δ*gloA* Δ*tpiA* were diluted 1:100-fold into fresh medium and exposed to ampicillin (200 µg/ml) for 20 h. After the treatment, cells were harvested, washed with 1× PBS, and plated on LB agar to count colony formation units (CFU). Plates were incubated for 72 h to allow small colonies to develop fully, and images were captured at specified time points. N=4.

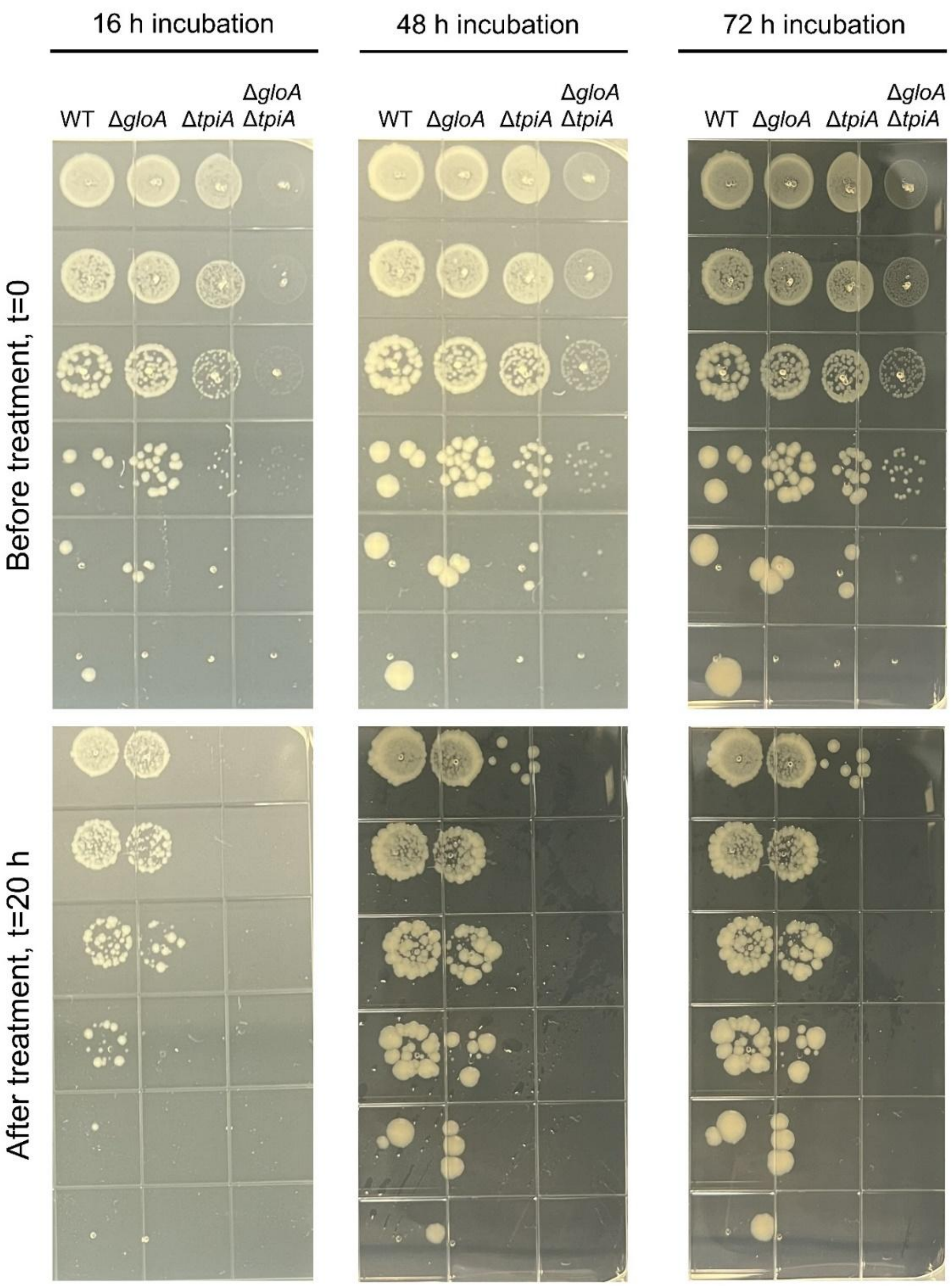

Figure S11. Colony formation of WT and mutant strains visualized on agar plates, related to Figure 5. Late-stationary-phase (24 h) cultures of *E. coli* WT, Δ*gloA*, Δ*tpiA*, and the double mutant Δ*gloA* Δ*tpiA* were diluted 1:100 into fresh medium and exposed to ofloxacin (5 µg/ml) for 20 h. After the treatment, cells were harvested, washed with 1× PBS, and plated on LB agar to count CFU. Plates were incubated for 72 h to allow small colonies to develop fully, and images were captured at specified time points. N=4.

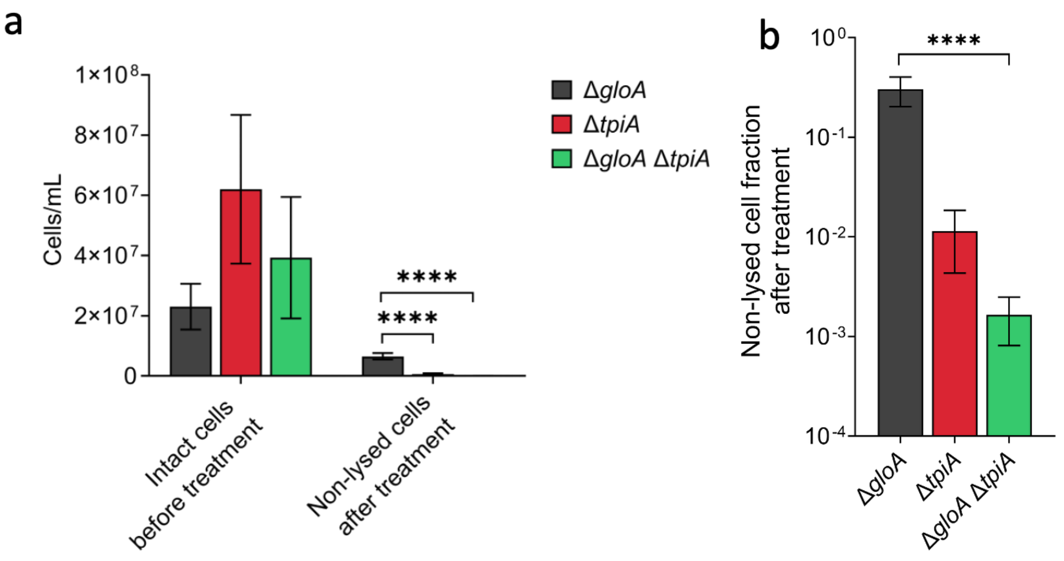

Figure S12. Flow cytometry quantification of intact cells after ampicillin treatment, related to Figure 7. Intact cell (GFP-positive) numbers were measured by flow cytometry before and after 20 h of ampicillin treatment in WT, Δ*gloA*, Δ*tpiA*, and Δ*gloA*Δ*tpiA* strains. (b) The intact cell fraction (non-lysed cell fraction) was determined as the ratio of intact cells after treatment to the initial cell number. The graph in panel (b) is displayed on a log scale. N = 4. Statistical analysis was performed using two-way ANOVA with Dunnett's multiple comparisons test; ****$P < 0.0001$. Data represent mean ± standard deviation from four independent biological replicates.

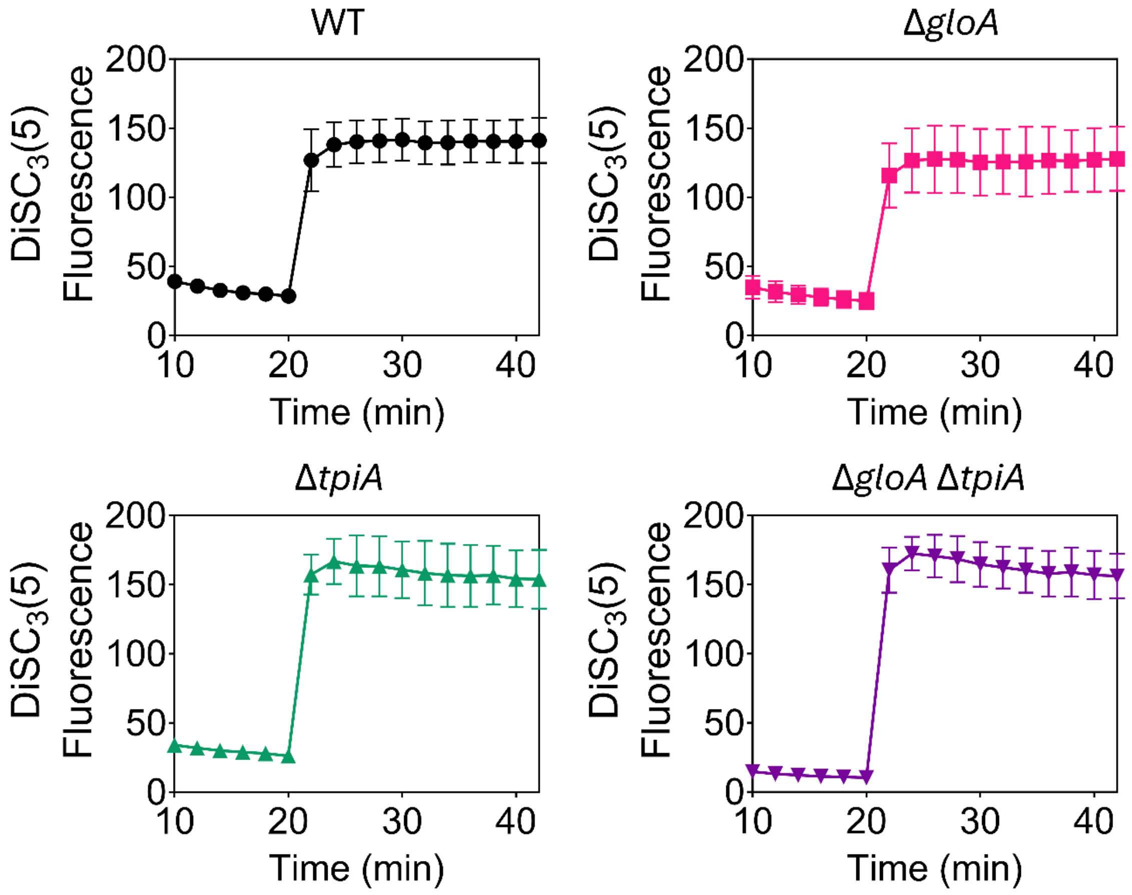

Figure S13. Control experiment for the DiSC$_3$(5) assay, related to Figure 8. Late stationary phase (24 h) cells of *E. coli* WT, Δ*gloA*, Δ*tpiA*, and the double mutant Δ*gloA* Δ*tpiA* were diluted to an OD_{600} of 0.1, washed with buffer containing 5 mM HEPES and 20 mM glucose, and stained with 1 μM DiSC$_3$(5) dye. After reaching equilibrium, polymyxin B was added, and fluorescence was measured at the indicated time points using a plate reader. Polymyxin B was used as a control to confirm proper dye function. N = 4. Data represent the mean ± standard deviation.

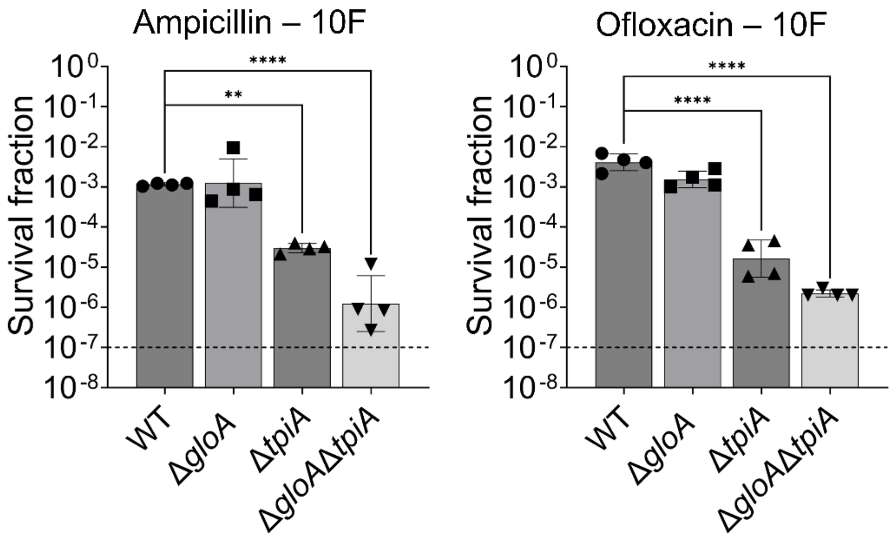

Figure S14. Assessment of survival fraction at increased cell density, related to Figure 8. *E. coli* WT and mutant strains (Δ*gloA*, Δ*tpiA*, and the double mutant Δ*gloA* Δ*tpiA*) at late stationary phase (24 h) were diluted 1:10-fold and immediately treated with ampicillin (200 µg/ml) and ofloxacin (5 µg/ml) for 20 h. After the treatment, cells were collected, washed with 1× PBS to remove the antibiotics and plated on LB agar to count CFU levels. N=4. Statistical analysis was performed between the WT and mutant strains using one-way ANOVA with Dunnett's post-test. *P<0.05, **P < 0.01, ***P < 0.001, and ****P < 0.0001. Data represent the mean ± standard deviation.

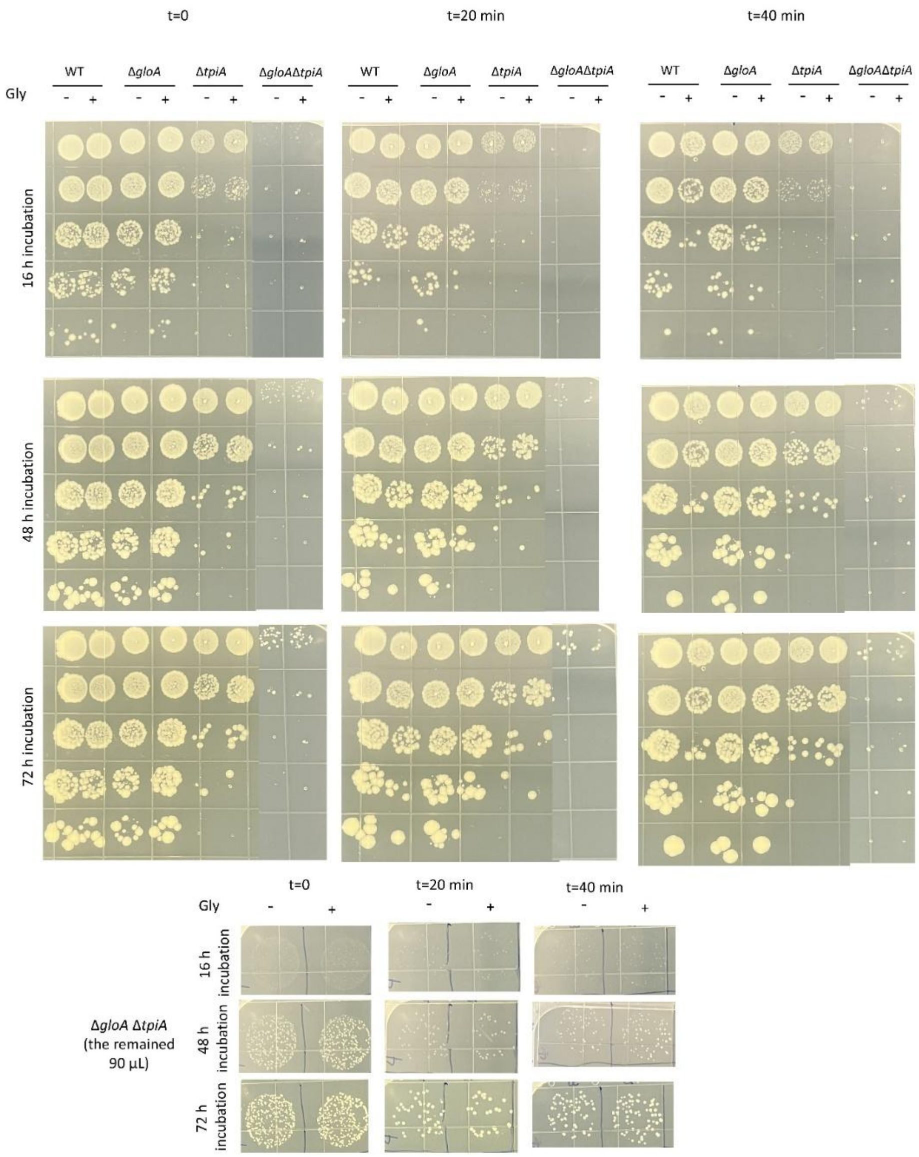

Figure S15. Colony formation resulting from the aminoglycoside potentiation assay for WT and mutant strains, visualized on agar plates, related to Figure 8. *E. coli* WT and mutant strains (Δ*gloA*, Δ*tpiA*, and the double mutant Δ*gloA* Δ*tpiA*) at late stationary phase (24 h) were diluted 1:10 and immediately exposed to **ampicillin** (200 µg/ml) for 20 hours. After treatment, surviving cells were collected, washed to remove residual antibiotics, and then treated with either DI water or 60 mM glycerol. Kanamycin (25 µg/ml) was subsequently added to designated cultures. At specified time points, 100 µL of cell suspension was collected, washed with 1× PBS, and resuspended in 100 µL of PBS. A 10 µL aliquot of each suspension was plated on LB agar to determine CFU levels. The remaining 90 µL of the double mutant suspension was also plated to increase the limit of CFU detection. Plates were incubated for 72 hours to allow full development of small colonies, and images were taken at the indicated time points. N=4.

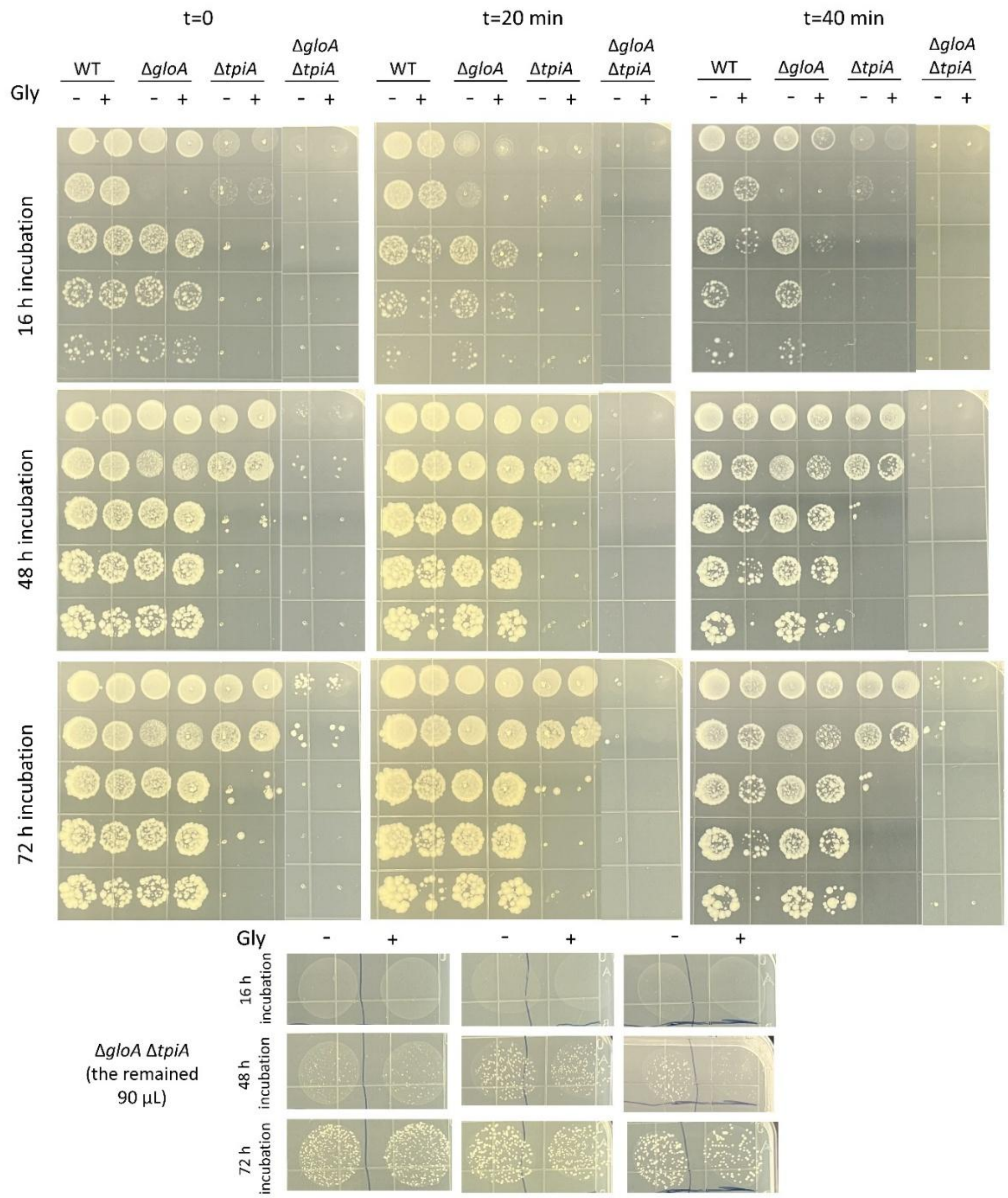

Figure S16. Colony formation resulting from the aminoglycoside potentiation assay for WT and mutant strains, visualized on agar plates, related to Figure 8. *E. coli* WT and mutant strains (Δ*gloA*, Δ*tpiA*, and the double mutant Δ*gloA* Δ*tpiA*) at late stationary phase (24 h) were diluted 1:10 and immediately exposed to **ofloxacin** (5 µg/ml) for 20 hours. After treatment, surviving cells were collected, washed to remove residual antibiotics, and then treated with either DI water or 60 mM glycerol. Kanamycin (25 µg/ml) was subsequently added to designated cultures. At specified time points, 100 µL of cell suspension was collected, washed with 1× PBS, and resuspended in 100 µL of PBS. A 10 µL aliquot of each suspension was plated on LB agar to determine CFU levels. The remaining 90 µL of the double mutant suspension was also plated to increase the limit of CFU detection. Plates were incubated for 72 hours to allow full development of small colonies, and images were taken at the indicated time points. N=4.

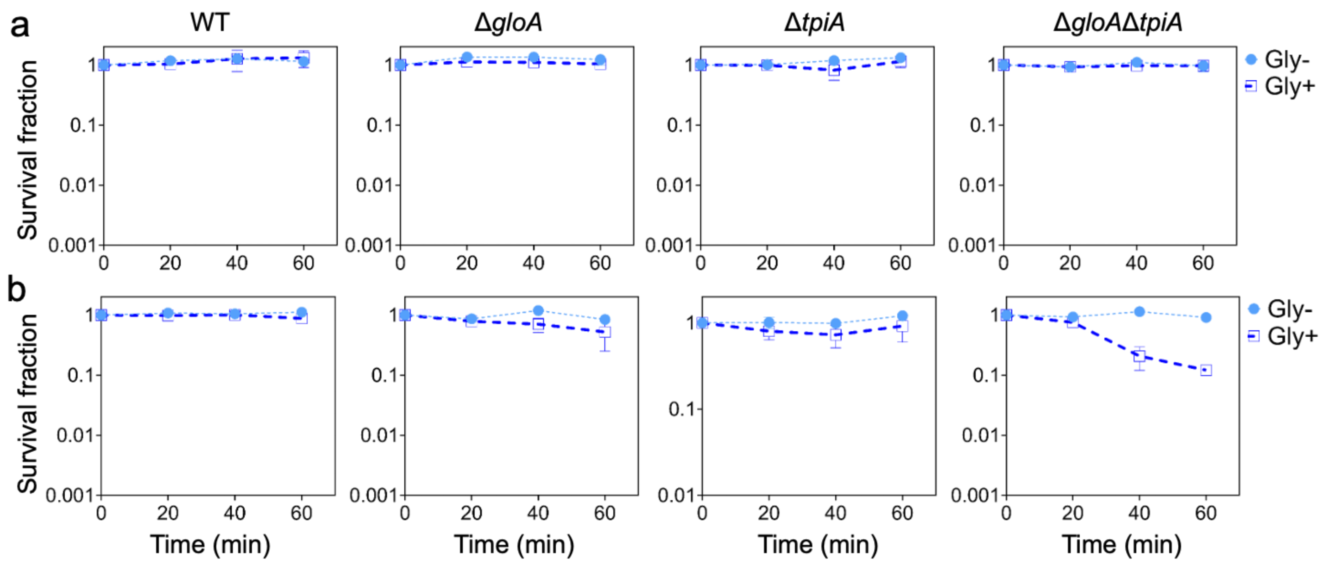

Figure S17. Recovery assays of antibiotic-tolerant persister cells in the absence of aminoglycosides, related to Figure 8. Late stationary-phase cultures were treated with ampicillin (panel a) or ofloxacin (panel b) for 20 h, then incubated in M9 minimal medium supplemented with either glycerol (Gly+) (60 mM) or deionized water (glycerol negative, Gly−) as a control. N = 4.

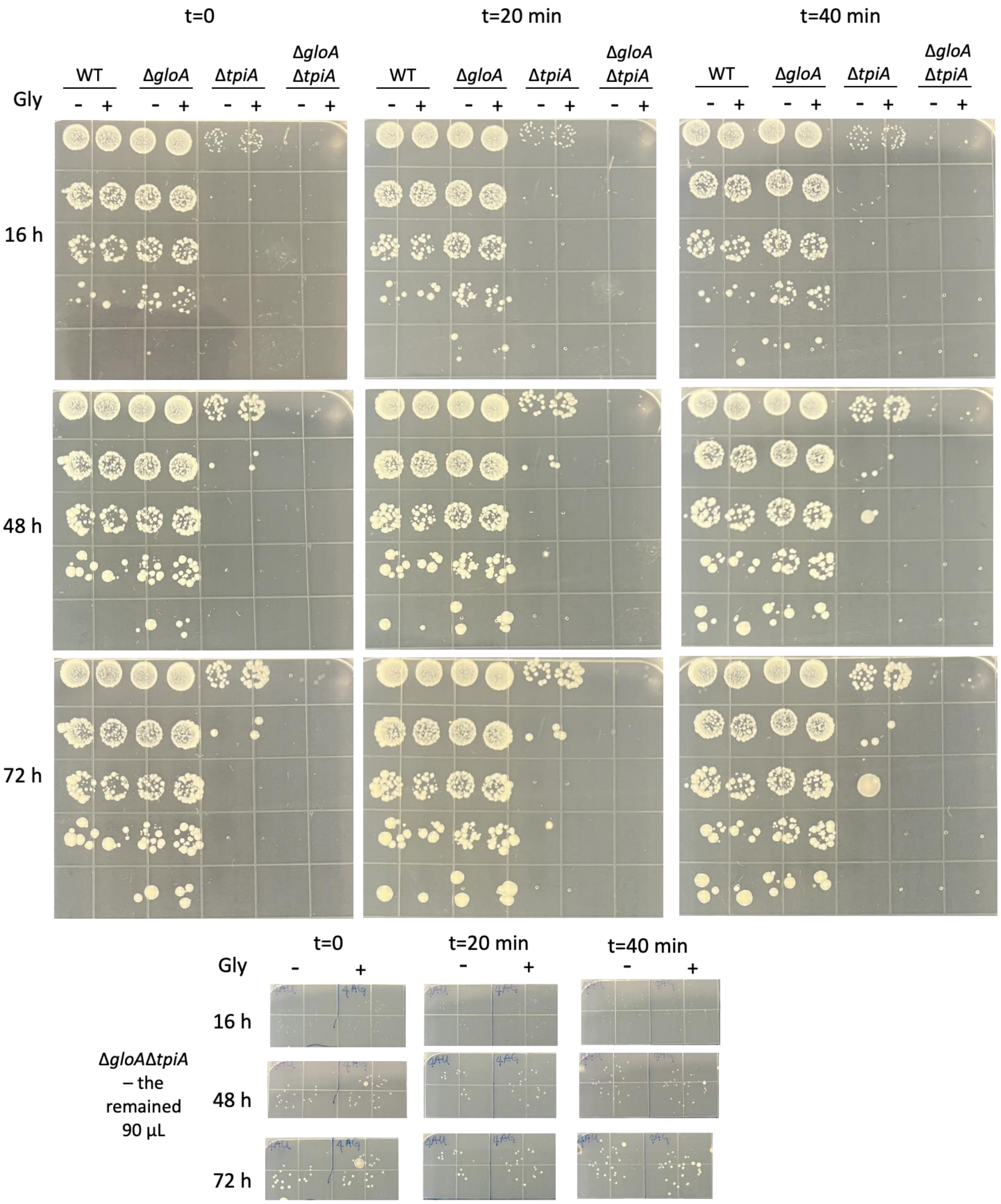

Figure S18. Colony formation from the recovery assays, visualized on agar plates, related to Figure 8. *E. coli* WT and mutant strains (Δ*gloA*, Δ*tpiA*, and the double mutant Δ*gloA* Δ*tpiA*) at late stationary phase (24 h) were diluted 1:10 and immediately exposed to **ampicillin** (200 µg/ml) for 20 hours. After treatment, surviving cells were collected, washed to remove residual antibiotics, and then treated with either DI water or 60 mM glycerol. **No kanamycin was added**. At specified time points, 100 µL of cell suspension was collected, washed with 1× PBS, and resuspended in 100 µL of PBS. A 10 µL aliquot of each suspension was plated on LB agar to determine CFU levels. The remaining 90 µL of the double mutant suspension was also plated to increase the limit of CFU detection. Plates were incubated for 72 hours to allow full development of small colonies, and images were taken at the indicated time points. N=4.

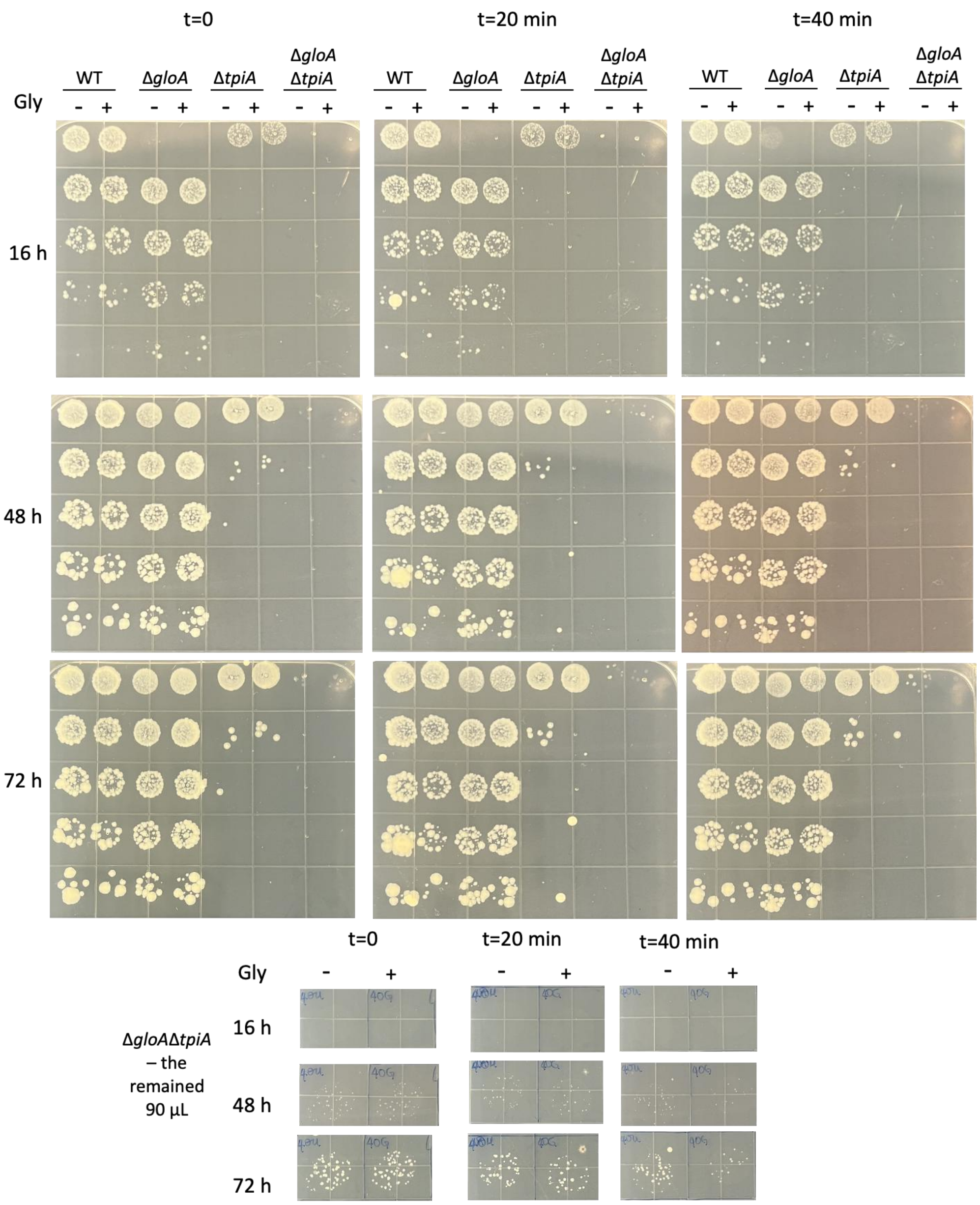

Figure S19. Colony formation from the recovery assays, visualized on agar plates, related to Figure 8. *E. coli* WT and mutant strains (Δ*gloA*, Δ*tpiA*, and the double mutant Δ*gloA* Δ*tpiA*) at late stationary phase (24 h) were diluted 1:10 and immediately exposed to **ofloxacin** (5 μg/ml) for 20 hours. After treatment, surviving cells were collected, washed to remove residual antibiotics, and then treated with either DI water or 60 mM glycerol. **No kanamycin was added**. At specified time points, 100 μL of cell suspension was collected, washed with 1× PBS, and resuspended in 100 μL of PBS. A 10 μL aliquot of each suspension was plated on LB agar to determine CFU levels. The remaining 90 μL of the double mutant suspension was also plated to increase the limit of CFU detection. Plates were incubated for 72 hours to allow full development of small colonies, and images were taken at the indicated time points. N=4.

Supplementary Tables

Table S2. Survival fractions of knockout strains following ampicillin and ofloxacin treatment, related to Figure 5.

KO strain	Ampicillin survival fraction		Ofloxacin survival fraction
ΔrbfA	2.94E-08		1.89E-06
ΔaspA	3.45E-08		1.52E-06
ΔlysS	3.70E-08		2.27E-07
Δefp	4.35E-08		9.33E-07
ΔselB	4.55E-08		1.74E-06
ΔglpT	5.26E-08		5.00E-07
ΔsdhA	1.25E-07		8.57E-07
ΔglpA	1.30E-07		9.13E-07
ΔcarB	1.67E-07		5.50E-06
ΔpolA	2.00E-07		7.37E-07
ΔyahK	3.87E-07		1.92E-06
ΔyhdE	8.15E-07		4.78E-07
ΔclpA	1.88E-06		3.83E-06
ΔompW	8.64E-06		4.40E-05
ΔgroL	1.14E-05		5.26E-05
ΔlacI	1.29E-05		9.67E-05
ΔasnB	1.36E-05		4.71E-07
ΔlacY	1.61E-05		5.60E-05
ΔyfcH	2.94E-05		6.00E-04
ΔdegP	5.71E-05		1.40E-04
ΔglpB	7.50E-05		5.56E-03
ΔnuoF	8.75E-05		2.11E-02
ΔackA	1.15E-04		1.14E-03
Δspy	1.92E-04		1.31E-03
Δfic	3.11E-04		1.82E-02
ΔdkgB	3.14E-04		5.13E-02
ΔfepB	5.67E-04		6.55E-02
Δgnd	6.36E-04		1.10E-02
ΔglpF	6.84E-04		8.89E-03
ΔilvN	7.33E-04		1.92E-02
ΔbtuE	1.06E-03		2.50E-02
ΔpykA	1.13E-03		7.22E-03
ΔldhA	1.13E-03		1.38E-02
ΔptsG	1.42E-03		1.81E-02

ΔiaaA	1.44E-03		2.20E-02
ΔompX	1.52E-03		9.00E-02
ΔpykF	1.67E-03		4.38E-02
ΔcomR	1.67E-03		2.10E-01
ΔgabD	1.83E-03		7.86E-03
ΔfrdB	1.93E-03		1.33E-02
ΔyqcC	3.13E-03		8.46E-02
Δmfd	3.50E-03		1.46E-02
ΔfrdA	3.53E-03		1.06E-02
Δndk	3.64E-03		4.07E-02
ΔydhS	3.79E-03		1.10E-01
ΔpoxB	4.17E-03		3.29E-02
ΔnuoE	4.50E-03		9.75E-03
ΔydcL	4.93E-03		8.67E-02
ΔarcB	5.22E-03		1.67E-01
ΔrclA	2.43E-02		5.40E-02

Table S3. Differential abundance of major *E. coli* phospholipid species and glycerol-3-phosphate in early and late stationary phase, related to Figure 5.

Wild-type *E. coli* cells were grown under stationary-phase culture conditions and harvested at early (t=5 h) and late (t=24 h) stationary phase. Metabolite and lipid abundances were quantified by LC–MS at Metabolon, Inc., and statistical significance was assessed using an ANOVA contrast for the early (ESP) versus late (LSP) stationary phase comparison; q-values reflect Benjamini–Hochberg correction for multiple testing. Note that phosphatidylethanolamine (PE) and phosphatidylglycerol (PG) species are the dominant *E. coli* membrane phospholipids and represent the most abundant potential sources of glycerol-3-phosphate generated through phospholipid turnover. Fold changes represent the LSP/ESP ratio, with values less than 1 indicating reduction during stationary-phase progression. Red: Significantly upregulated; green: significantly downregulated. Raw data for this analysis are available in the Reference [54].

Sub-pathway	Biochemical name	LSP/ESP	p-value	q-value
Phosphatidylethanolamine (PE)	1,2-dipalmitoyl-GPE (16:0/16:0)	**1.58**	0.00010000	0.00010000
	1-palmitoyl-2-stearoyl-GPE (16:0/18:0)	**0.75**	0.02300000	0.01010000
	1-palmitoyl-2-oleoyl-GPE (16:0/18:1)	**0.07**	0.00000002	0.00000005
	1-palmitoleoyl-2-oleoyl-GPE (16:1/18:1)	**0.02**	0.00000000	0.00000001
	1-stearoyl-2-oleoyl-GPE (18:0/18:1)	**0.21**	0.00000039	0.00000072
	1-stearoyl-2-linoleoyl-GPE (18:0/18:2)	**3.03**	0.00005060	0.00004462
	1,2-dioleoyl-GPE (18:1/18:1)	**0.03**	0.00000001	0.00000003
Phosphatidylglycerol (PG)	1,2-dipalmitoyl-GPG (16:0/16:0)	**1.76**	0.00020000	0.00020000
	1-palmitoyl-2-palmitoleoyl-GPG (16:0/16:1)	**0.25**	0.00000139	0.00000200
	1-palmitoyl-2-oleoyl-GPG (16:0/18:1)	**0.53**	0.00020000	0.00010000
	1-palmitoleoyl-2-oleoyl-GPG (16:1/18:1)	**0.07**	0.00000003	0.00000008
	1,2-dioleoyl-GPG (18:1/18:1)	**0.12**	0.00000019	0.00000039
Lysophospholipid	1-palmitoyl-GPA (16:0)	1.09	0.99650000	0.27890000
	1-palmitoleoyl-GPA (16:1)	**0.28**	0.00080000	0.00050000
	1-palmitoyl-GPE (16:0)	**0.23**	0.00000324	0.00000415
	1-stearoyl-GPE (18:0)	**0.44**	0.00040000	0.00030000
	2-stearoyl-GPE (18:0)	0.49	0.15150000	0.05360000
	1-oleoyl-GPE (18:1)	**0.03**	0.00000001	0.00000003
	1-palmitoyl-GPG (16:0)	**0.14**	0.00009467	0.00007891
	1-stearoyl-GPG (18:0)	1.33	0.49060000	0.15460000
	1-oleoyl-GPG (18:1)	**0.03**	0.00000000	0.00000001
Glycerolipid Metabolism	**glycerol 3-phosphate**	**0.33**	0.00080000	0.00050000

Table S4. Differential abundance of major *E. coli* phospholipid species and glycerol-3-phosphate in phenothiazine-treated stationary-phase cells, related to Figure 5.

Wild-type *E. coli* cells were grown under stationary-phase culture conditions, treated with thioridazine (TDZ) at 5 h of growth, and harvested for metabolomic analysis at 24 h. Metabolite and lipid abundances were quantified by LC–MS at Metabolon, Inc., and statistical significance for TDZ-treated versus untreated samples was assessed using the Welch two-sample t-test; q-values reflect Benjamini–Hochberg correction for multiple testing. PE and PG components are the dominant *E. coli* membrane phospholipids and represent the most abundant potential sources of glycerol-3-phosphate generated through phospholipid turnover. Fold changes represent the ratio of TDZ-treated to untreated cells, with values greater than 1 indicating accumulation under PMF-inhibitory conditions. Red: Significantly upregulated; green: significantly downregulated. Raw data for this analysis are available in the Reference [55].

Note: Phenothiazine treatment is relevant because these compounds collapse PMF and suppress TCA and ETC activity, creating a defined metabolic state similar to that of our respiratory-deficient mutants. The lipid data associated with this treatment also provide a practical advantage, as they allow assessment of PMF-dependent effects on phospholipid turnover and glycerol-3-phosphate metabolism without requiring exhaustive testing across all metabolic mutants.

Sub-pathway	Biochemical name	TDZ/ Untreated	p-value	q-value
Phosphatidylethanolamine (PE)	1,2-dipalmitoyl-GPE (16:0/16:0)	**0.76**	0.24360000	0.10070000
	1-palmitoyl-2-stearoyl-GPE (16:0/18:0)	**2.43**	0.00300000	0.00190000
	1-palmitoyl-2-oleoyl-GPE (16:0/18:1)	**16.62**	0.00030000	0.00020000
	1-palmitoleoyl-2-oleoyl-GPE (16:1/18:1)	**54.02**	0.07120000	0.03290000
	1-stearoyl-2-oleoyl-GPE (18:0/18:1)	**7.86**	0.00040000	0.00030000
	1-stearoyl-2-linoleoyl-GPE (18:0/18:2)	**0.33**	0.00020000	0.00020000
	1,2-dioleoyl-GPE (18:1/18:1)	**40.18**	0.00450000	0.00270000
Phosphatidylglycerol (PG)	1,2-dipalmitoyl-GPG (16:0/16:0)	**0.56**	0.00070000	0.00050000
	1-palmitoyl-2-palmitoleoyl-GPG (16:0/16:1)	**4.42**	0.06970000	0.03240000
	1-palmitoyl-2-oleoyl-GPG (16:0/18:1)	**2.38**	0.00440000	0.00270000
	1-palmitoleoyl-2-oleoyl-GPG (16:1/18:1)	**16.01**	0.00040000	0.00030000
	1,2-dioleoyl-GPG (18:1/18:1)	**11.52**	0.75220000	0.26860000
Lysophospholipid	1-palmitoyl-GPA (16:0)	2.88	0.23210000	0.09690000
	1-palmitoleoyl-GPA (16:1)	**22.09**	1.00000000	0.33270000
	1-palmitoyl-GPE (16:0)	**9.17**	0.00780000	0.00450000
	1-stearoyl-GPE (18:0)	**9.80**	0.00002107	0.00002398
	2-stearoyl-GPE (18:0)	1.35	0.40530000	0.15810000
	1-oleoyl-GPE (18:1)	**62.35**	0.00290000	0.00180000
	1-palmitoyl-GPG (16:0)	3.32	0.37180000	0.14600000
	1-stearoyl-GPG (18:0)	1.19	0.00050000	0.00040000
	1-oleoyl-GPG (18:1)	**66.90**	0.00004725	0.00004777
Glycerolipid Metabolism	**glycerol 3-phosphate**	**6.38**	0.00000732	0.00000944

Table S5. Concentrations of bactericidal antibiotics used in persister assays, related to Figure 5.

	**Concentration (μg/mL)**	
**Bacterial Strains**	**Ampicillin**	**Ofloxacin**
MIC of *E. coli* K-12 MG1655 Wild Type	1.5-2	0.032-0.047
MIC of *E. coli* K-12 MG1655 Δ*gloA*	1.5-2	0.032-0.047
MIC of *E. coli* K-12 MG1655 Δ*tpiA*	1.5-2	0.047-0.064
MIC of *E. coli* K-12 MG1655 Δ*gloA* Δ*tpiA*	3-4	0.094-0.125
Persister Assay Concentration	200	5

Table S6. Bacterial strains and plasmids used in this study, related to STAR Methods.

Bacterial Strains	Source
Escherichia coli K-12 MG1655 Wild Type	Gift from Dr. Mark P. Brynildsen
Escherichia coli K-12 BW25113 Wild Type	Keio collection, Cat# OEC4988
Escherichia coli K-12 MG1655 Δ*gloA*	This study
Escherichia coli K-12 MG1655 ΔtpiA	This study
Escherichia coli K-12 MG1655 ΔgloAΔ*tpiA*	This study
Escherichia coli K-12 BW25113 Δ*rbfA*	Keio collection, Cat# OEC4988
Escherichia coli K-12 BW25113 Δ*aspA*	Keio collection, Cat# OEC4988
Escherichia coli K-12 BW25113 Δ*lysS*	Keio collection, Cat# OEC4988
Escherichia coli K-12 BW25113 Δ*efp*	Keio collection, Cat# OEC4988
Escherichia coli K-12 BW25113 Δ*selB*	Keio collection, Cat# OEC4988
Escherichia coli K-12 BW25113 Δ*glpT*	Keio collection, Cat# OEC4988
Escherichia coli K-12 BW25113 Δ*sdhA*	Keio collection, Cat# OEC4988
Escherichia coli K-12 BW25113 Δ*glpA*	Keio collection, Cat# OEC4988
Escherichia coli K-12 BW25113 Δ*carB*	Keio collection, Cat# OEC4988
Escherichia coli K-12 BW25113 Δ*polA*	Keio collection, Cat# OEC4988
Escherichia coli K-12 BW25113 Δ*yahK*	Keio collection, Cat# OEC4988
Escherichia coli K-12 BW25113 Δ*yhdE*	Keio collection, Cat# OEC4988
Escherichia coli K-12 BW25113 Δ*clpA*	Keio collection, Cat# OEC4988
Escherichia coli K-12 BW25113 Δ*ompW*	Keio collection, Cat# OEC4988
Escherichia coli K-12 BW25113 Δ*groL*	Keio collection, Cat# OEC4988
Escherichia coli K-12 BW25113 Δ*lacI*	Keio collection, Cat# OEC4988
Escherichia coli K-12 BW25113 Δ*asnB*	Keio collection, Cat# OEC4988
Escherichia coli K-12 BW25113 Δ*lacY*	Keio collection, Cat# OEC4988
Escherichia coli K-12 BW25113 Δ*yfcH*	Keio collection, Cat# OEC4988
Escherichia coli K-12 BW25113 Δ*degP*	Keio collection, Cat# OEC4988
Escherichia coli K-12 BW25113 Δ*glpB*	Keio collection, Cat# OEC4988
Escherichia coli K-12 BW25113 Δ*nuoF*	Keio collection, Cat# OEC4988
Escherichia coli K-12 BW25113 Δ*ackA*	Keio collection, Cat# OEC4988
Escherichia coli K-12 BW25113 Δ*spy*	Keio collection, Cat# OEC4988
Escherichia coli K-12 BW25113 Δ*fic*	Keio collection, Cat# OEC4988
Escherichia coli K-12 BW25113 Δ*dkgB*	Keio collection, Cat# OEC4988
Escherichia coli K-12 BW25113 Δ*fepB*	Keio collection, Cat# OEC4988
Escherichia coli K-12 BW25113 Δ*gnd*	Keio collection, Cat# OEC4988
Escherichia coli K-12 BW25113 Δ*glpF*	Keio collection, Cat# OEC4988
Escherichia coli K-12 BW25113 Δ*ilvN*	Keio collection, Cat# OEC4988
Escherichia coli K-12 BW25113 Δ*btuE*	Keio collection, Cat# OEC4988
Escherichia coli K-12 BW25113 Δ*pykA*	Keio collection, Cat# OEC4988
Escherichia coli K-12 BW25113 Δ*ldhA*	Keio collection, Cat# OEC4988
Escherichia coli K-12 BW25113 Δ*ptsG*	Keio collection, Cat# OEC4988

Escherichia coli K-12 BW25113 Δ*iaaA*	Keio collection, Cat# OEC4988
Escherichia coli K-12 BW25113 Δ*ompX*	Keio collection, Cat# OEC4988
Escherichia coli K-12 BW25113 Δ*pykF*	Keio collection, Cat# OEC4988
Escherichia coli K-12 BW25113 Δ*comR*	Keio collection, Cat# OEC4988
Escherichia coli K-12 BW25113 Δ*gabD*	Keio collection, Cat# OEC4988
Escherichia coli K-12 BW25113 Δ*frdB*	Keio collection, Cat# OEC4988
Escherichia coli K-12 BW25113 Δ*yqcC*	Keio collection, Cat# OEC4988
Escherichia coli K-12 BW25113 Δ*mfd*	Keio collection, Cat# OEC4988
Escherichia coli K-12 BW25113 Δ*frdA*	Keio collection, Cat# OEC4988
Escherichia coli K-12 BW25113 Δ*ndk*	Keio collection, Cat# OEC4988
Escherichia coli K-12 BW25113 Δ*ydhS*	Keio collection, Cat# OEC4988
Escherichia coli K-12 BW25113 Δ*poxB*	Keio collection, Cat# OEC4988
Escherichia coli K-12 BW25113 Δ*nuoE*	Keio collection, Cat# OEC4988
Escherichia coli K-12 BW25113 Δ*ydcL*	Keio collection, Cat# OEC4988
Escherichia coli K-12 BW25113 Δ*arcB*	Keio collection, Cat# OEC4988
Escherichia coli K-12 BW25113 Δ*rclA*	Keio collection, Cat# OEC4988
**Bacterial Plasmids**	**Source or Reference**
pUA66-*ftsZ*-*gfp*	Previous study (Mohiuddin 2022)

Table S7. Oligonucleotides for the generation and verification of mutant strains, related to STAR Methods.

**Oligonucleotides to Generate Gene Deletions**					
**Mutation**	**Forward Primer (5' to 3')**	**Reverse Primer (5' to 3')**	**Source**		
Δ*gloA::Kan*	CGCTATACTAAAACAACATTTTGAATCTGTTAGCCATTTTGAGGATAAAAAGGTGTAGGCTGGAGCTGCTTC	GTAAAGATGCGGGCGCGATGAGTTCACGCCCGGCAGGAGATTAACGGCTGACATGGGAAT	Integrated DNA Technologies, Inc.		
Δ*tpiA::Kan*	GCCATCTTCCTTTATTCGCTTATAAGCGTGGAGAATTAAAGTGTAGGCTGGAGCTGCTTC	GAAAGTAAGTGCCGGATATGAAATCCGGCACCTGTCAGACTTAACGGCTGACATGGGAAT	Integrated DNA Technologies, Inc.		
**Oligonucleotides to Verify Gene Deletions**					
**Mutation**	**External Forward Primer (5' to 3')**	**External Reverse Primer (5' to 3')**	**Internal Forward Primer (5' to 3')**	**Internal Reverse Primer (5' to 3')**	**Source**
Δ*gloA::Kan*	TATACCGATTACCCGACGTT	GCTCTTCGTCCAGATCATCC	TTCATACCATGCTGCGCGTT	GACCGGCGTCTTTCTCTTCG	Integrated DNA Technologies, Inc.
Δ*tpiA::Kan*	CGCTGTTGAACCGATTAAGC	GCTCTTCGTCCAGATCATCC	AATCGCACCACCGGAAATGT	AAGCCTGTTTAGCCGCTTCTG	Integrated DNA Technologies, Inc.

Supplemental References

[1] I. Duarte-Velázquez *et al.*, 'Escherichia coli transcription factors of unknown function: sequence features and possible evolutionary relationships.', *PeerJ*, vol. 10, p. e13772, 2022, doi: 10.7717/peerj.13772.

[2] M. Mermod, D. Magnani, M. Solioz, and J. V Stoyanov, 'The copper-inducible ComR (YcfQ) repressor regulates expression of ComC (YcfR), which affects copper permeability of the outer membrane of Escherichia coli.', *Biometals*, vol. 25, no. 1, pp. 33–43, Feb. 2012, doi: 10.1007/s10534-011-9510-x.

[3] J. Jin *et al.*, 'Insights into the cellular function of YhdE, a nucleotide pyrophosphatase from Escherichia coli.', *PLoS One*, vol. 10, no. 2, p. e0117823, 2015, doi: 10.1371/journal.pone.0117823.

[4] A. Tchigvintsev *et al.*, 'Biochemical and structural studies of conserved Maf proteins revealed nucleotide pyrophosphatases with a preference for modified nucleotides.', *Chem. Biol.*, vol. 20, no. 11, pp. 1386–98, Nov. 2013, doi: 10.1016/j.chembiol.2013.09.011.

[5] K. Otto and M. Hermansson, 'Inactivation of ompX causes increased interactions of type 1 fimbriated Escherichia coli with abiotic surfaces.', *J. Bacteriol.*, vol. 186, no. 1, pp. 226–34, Jan. 2004, doi: 10.1128/JB.186.1.226-234.2004.

[6] K. Otto, J. Norbeck, T. Larsson, K. A. Karlsson, and M. Hermansson, 'Adhesion of type 1-fimbriated Escherichia coli to abiotic surfaces leads to altered composition of outer membrane proteins.', *J. Bacteriol.*, vol. 183, no. 8, pp. 2445–53, Apr. 2001, doi: 10.1128/JB.183.8.2445-2453.2001.

[7] A. S. Juncker, H. Willenbrock, G. Von Heijne, S. Brunak, H. Nielsen, and A. Krogh, 'Prediction of lipoprotein signal peptides in Gram-negative bacteria.', *Protein Sci.*, vol. 12, no. 8, pp. 1652–62, Aug. 2003, doi: 10.1110/ps.0303703.

[8] S. Iuchi and E. C. Lin, 'Mutational analysis of signal transduction by ArcB, a membrane sensor protein responsible for anaerobic repression of operons involved in the central aerobic pathways in Escherichia coli.', *J. Bacteriol.*, vol. 174, no. 12, pp. 3972–80, Jun. 1992, doi: 10.1128/jb.174.12.3972-3980.1992.

[9] S. Iuchi, D. C. Cameron, and E. C. Lin, 'A second global regulator gene (arcB) mediating repression of enzymes in aerobic pathways of Escherichia coli.', *J. Bacteriol.*, vol. 171, no. 2, pp. 868–73, Feb. 1989, doi: 10.1128/jb.171.2.868-873.1989.

[10] M. P. Roisin and A. Kepes, 'Nucleosidediphosphate kinase of Escherichia coli, a periplasmic enzyme.', *Biochim. Biophys. Acta*, vol. 526, no. 2, pp. 418–28, Oct. 1978, doi: 10.1016/0005-2744(78)90133-x.

[11] R. Utsumi, Y. Nakamoto, M. Kawamukai, M. Himeno, and T. Komano, 'Involvement of cyclic AMP and its receptor protein in filamentation of an Escherichia coli fic mutant.', *J. Bacteriol.*, vol. 151, no. 2, pp. 807–12, Aug. 1982, doi: 10.1128/jb.151.2.807-812.1982.

[12] J. D. Meredith, I. Chapman, K. Ulrich, C. Sebastian, F. Stull, and M. J. Gray, 'Escherichia coli RclA is a highly active hypothiocyanite reductase.', *Proc. Natl. Acad. Sci. U. S. A.*, vol. 119, no. 30, p. e2119368119, Jul. 2022, doi: 10.1073/pnas.2119368119.

[13] R. M. Derke *et al.*, ‘The Cu(II) Reductase RclA Protects Escherichia coli against the Combination of Hypochlorous Acid and Intracellular Copper.’, *mBio*, vol. 11, no. 5, Sep. 2020, doi: 10.1128/mBio.01905-20.

[14] Y. Baek *et al.*, ‘Structure and function of the hypochlorous acid-induced flavoprotein RclA from Escherichia coli.’, *J. Biol. Chem.*, vol. 295, no. 10, pp. 3202–3212, Mar. 2020, doi: 10.1074/jbc.RA119.011530.

[15] C. Sprencel *et al.*, ‘Binding of ferric enterobactin by the Escherichia coli periplasmic protein FepB.’, *J. Bacteriol.*, vol. 182, no. 19, pp. 5359–64, Oct. 2000, doi: 10.1128/JB.182.19.5359-5364.2000.

[16] F. A. Arenas, W. A. Díaz, C. A. Leal, J. M. Pérez-Donoso, J. A. Imlay, and C. C. Vásquez, ‘The Escherichia coli btuE gene, encodes a glutathione peroxidase that is induced under oxidative stress conditions.’, *Biochem. Biophys. Res. Commun.*, vol. 398, no. 4, pp. 690–4, Aug. 2010, doi: 10.1016/j.bbrc.2010.07.002.

[17] L. P. Freedman, J. M. Zengel, R. H. Archer, and L. Lindahl, ‘Autogenous control of the S10 ribosomal protein operon of Escherichia coli: genetic dissection of transcriptional and posttranscriptional regulation.’, *Proc. Natl. Acad. Sci. U. S. A.*, vol. 84, no. 18, pp. 6516–20, Sep. 1987, doi: 10.1073/pnas.84.18.6516.

[18] I. M. Keseler *et al.*, ‘EcoCyc: a comprehensive database of Escherichia coli biology’, *Nucleic Acids Res.*, vol. 39, no. Database, pp. D583–D590, Jan. 2011, doi: 10.1093/nar/gkq1143.

[19] E. Di Luccio, R. A. Elling, and D. K. Wilson, ‘Identification of a novel NADH-specific aldo-keto reductase using sequence and structural homologies.’, *Biochem. J.*, vol. 400, no. 1, pp. 105–14, Nov. 2006, doi: 10.1042/BJ20060660.

[20] A. Pick, B. Rühmann, J. Schmid, and V. Sieber, ‘Novel CAD-like enzymes from Escherichia coli K-12 as additional tools in chemical production.’, *Appl. Microbiol. Biotechnol.*, vol. 97, no. 13, pp. 5815–24, Jul. 2013, doi: 10.1007/s00253-012-4474-5.

[21] C. P. Selby, E. M. Witkin, and A. Sancar, ‘Escherichia coli mfd mutant deficient in “mutation frequency decline” lacks strand-specific repair: in vitro complementation with purified coupling factor.’, *Proc. Natl. Acad. Sci. U. S. A.*, vol. 88, no. 24, pp. 11574–8, Dec. 1991, doi: 10.1073/pnas.88.24.11574.

[22] O. V Sergeeva, D. O. Bredikhin, M. V Nesterchuk, M. V Serebryakova, P. V Sergiev, and O. A. Dontsova, ‘Possible Role of Escherichia coli Protein YbgI.’, *Biochemistry (Mosc).*, vol. 83, no. 3, pp. 270–280, Mar. 2018, doi: 10.1134/S0006297918030070.

[23] W. S. Yew and J. A. Gerlt, ‘Utilization of L-ascorbate by Escherichia coli K-12: assignments of functions to products of the yjf-sga and yia-sgb operons.’, *J. Bacteriol.*, vol. 184, no. 1, pp. 302–6, Jan. 2002, doi: 10.1128/JB.184.1.302-306.2002.

[24] M. Yamamoto, M. Nomura, H. Ohsawa, and B. Maruo, ‘Identification of a temperature-sensitive asparaginyl-transfer ribonucleic acid synthetase mutant of Escherichia coli.’, *J. Bacteriol.*, vol. 132, no. 1, pp. 127–31, Oct. 1977, doi: 10.1128/jb.132.1.127-131.1977.

[25] S. T. Cole, T. Grundström, B. Jaurin, J. J. Robinson, and J. H. Weiner, ‘Location and nucleotide sequence of frdB, the gene coding for the iron-sulphur protein subunit of the fumarate reductase of Escherichia coli.’, *Eur. J. Biochem.*, vol. 126, no. 1, pp. 211–6, Aug. 1982, doi: 10.1111/j.1432-1033.1982.tb06768.x.

[26] A. J. Sussman and C. Gilvarg, ‘Peptidases in Escherichia coli K-12 capable of cleaving lysine homopeptides.’, *J. Biol. Chem.*, vol. 245, no. 24, pp. 6518–24, Dec. 1970, doi: 10.1016/S0021-9258(18)62564-5.

[27] G. Eriani, G. Dirheimer, and J. Gangloff, ‘Aspartyl-tRNA synthetase from Escherichia coli: cloning and characterisation of the gene, homologies of its translated amino acid sequence with asparaginyl- and lysyl-tRNA synthetases.’, *Nucleic Acids Res.*, vol. 18, no. 23, pp. 7109–18, Dec. 1990, doi: 10.1093/nar/18.23.7109.

[28] F. Stenberg *et al.*, ‘Protein complexes of the Escherichia coli cell envelope.’, *J. Biol. Chem.*, vol. 280, no. 41, pp. 34409–19, Oct. 2005, doi: 10.1074/jbc.M506479200.

[29] R. P. Holmes and R. R. Russell, ‘Mutations affecting amino sugar metabolism in Escherichia coli K-12.’, *J. Bacteriol.*, vol. 111, no. 1, pp. 290–1, Jul. 1972, doi: 10.1128/jb.111.1.290-291.1972.

[30] H. Bergler *et al.*, ‘Protein EnvM is the NADH-dependent enoyl-ACP reductase (FabI) of Escherichia coli.’, *J. Biol. Chem.*, vol. 269, no. 8, pp. 5493–6, Feb. 1994, doi: 10.1016/S0021-9258(17)37485-9.

[31] S. T. Cole, ‘Nucleotide sequence coding for the flavoprotein subunit of the fumarate reductase of Escherichia coli.’, *Eur. J. Biochem.*, vol. 122, no. 3, pp. 479–84, Mar. 1982, doi: 10.1111/j.1432-1033.1982.tb06462.x.

[32] I. Cozzani, A. M. Fazio, E. Felici, and G. Barletta, ‘Separation and characterization of NAD- and NADP-specific succinate-semialdehyde dehydrogenase from Escherichia coli K-12 3300.’, *Biochim. Biophys. Acta*, vol. 613, no. 2, pp. 309–17, Jun. 1980, doi: 10.1016/0005-2744(80)90085-6.

[33] H. L. Kornberg and M. Malcovati, ‘Control in situ of the pyruvate kinase activity of Escherichia coli.’, *FEBS Lett.*, vol. 32, no. 2, pp. 257–9, Jun. 1973, doi: 10.1016/0014-5793(73)80846-4.

[34] S. T. Cole *et al.*, ‘Nucleotide sequence and gene-polypeptide relationships of the glpABC operon encoding the anaerobic sn-glycerol-3-phosphate dehydrogenase of Escherichia coli K-12.’, *J. Bacteriol.*, vol. 170, no. 6, pp. 2448–56, Jun. 1988, doi: 10.1128/jb.170.6.2448-2456.1988.

[35] A. Schryvers and J. H. Weiner, ‘The anaerobic sn-glycerol-3-phosphate dehydrogenase: cloning and expression of the glpA gene of Escherichia coli and identification of the glpA products.’, *Can. J. Biochem.*, vol. 60, no. 3, pp. 224–31, Mar. 1982, doi: 10.1139/o82-027.

[36] R. Lim and S. S. Cohen, ‘D-phosphoarabinoisomerase and D-ribulokinase in Escherichia coli.’, *J. Biol. Chem.*, vol. 241, no. 19, pp. 4304–15, Oct. 1966, doi: 10.1016/S0021-9258(18)99723-1.

[37] K. L. Strauch, K. Johnson, and J. Beckwith, ‘Characterization of degP, a gene required for proteolysis in the cell envelope and essential for growth of Escherichia coli at high temperature.’, *J. Bacteriol.*, vol. 171, no. 5, pp. 2689–96, May 1989, doi: 10.1128/jb.171.5.2689-2696.1989.

[38] F. B. Rudolph and H. J. Fromm, ‘The purification and properties of aspartase from Escherichia coli.’, *Arch. Biochem. Biophys.*, vol. 147, no. 1, pp. 92–8, Nov. 1971, doi: 10.1016/0003-9861(71)90313-4.

[39] E. M. Tarmy and N. O. Kaplan, ‘Kinetics of Escherichia coli B D-lactate dehydrogenase and evidence for pyruvate-controlled change in conformation.’, *J. Biol. Chem.*, vol. 243, no. 10, pp. 2587–96, May 1968, doi: 10.1016/S0021-9258(18)93414-9.

[40] S. Dover and Y. S. Halpern, ‘Genetic analysis of the gamma-aminobutyrate utilization pathway in Escherichia coli K-12.’, *J. Bacteriol.*, vol. 117, no. 2, pp. 494–501, Feb. 1974, doi: 10.1128/jb.117.2.494-501.1974.

[41] D. G. Fraenkel, ‘Selection of Escherichia coli mutants lacking glucose-6-phosphate dehydrogenase or gluconate-6-phosphate dehydrogenase.’, *J. Bacteriol.*, vol. 95, no. 4, pp. 1267–71, Apr. 1968, doi: 10.1128/jb.95.4.1267-1271.1968.

[42] G. A. O’Donovan, G. Edlin, J. A. Fuchs, J. Neuhard, and E. Thomassen, ‘Deoxycytidine triphosphate deaminase: characterization of an Escherichia coli mutant deficient in the enzyme.’, *J. Bacteriol.*, vol. 105, no. 2, pp. 666–72, Feb. 1971, doi: 10.1128/jb.105.2.666-672.1971.

[43] R. J. White, ‘The role of the phosphoenolpyruvate phosphotransferase system in the transport of N-acetyl-D-glucosamine by Escherichia coli.’, *Biochem. J.*, vol. 118, no. 1, pp. 89–92, Jun. 1970, doi: 10.1042/bj1180089.

[44] A. Săsărman, P. Chartrand, R. Proschek, M. Desrochers, D. Tardif, and C. Lapointe, ‘Uroporphyrin-accumulating mutant of Escherichia coli K-12.’, *J. Bacteriol.*, vol. 124, no. 3, pp. 1205–12, Dec. 1975, doi: 10.1128/jb.124.3.1205-1212.1975.

[45] A. M. Abdel-Hamid, M. M. Attwood, and J. R. Guest, ‘Pyruvate oxidase contributes to the aerobic growth efficiency of Escherichia coli.’, *Microbiology (Reading)*, vol. 147, no. Pt 6, pp. 1483–1498, Jun. 2001, doi: 10.1099/00221287-147-6-1483.

[46] N. Imamura and H. Nakayama, ‘thiK and thiL loci of Escherichia coli.’, *J. Bacteriol.*, vol. 151, no. 2, pp. 708–17, Aug. 1982, doi: 10.1128/jb.151.2.708-717.1982.

[47] A. Saini, D. T. Mapolelo, H. K. Chahal, M. K. Johnson, and F. W. Outten, ‘SufD and SufC ATPase activity are required for iron acquisition during in vivo Fe-S cluster formation on SufB.’, *Biochemistry*, vol. 49, no. 43, pp. 9402–12, Nov. 2010, doi: 10.1021/bi1011546.

[48] C. Saveanu *et al.*, ‘Structural and nucleotide-binding properties of YajQ and YnaF, two Escherichia coli proteins of unknown function.’, *Protein Sci.*, vol. 11, no. 11, pp. 2551–60, Nov. 2002, doi: 10.1110/ps.0217502.

[49] J.-W. Choi, J. Lee, K. Nishi, Y.-S. Kim, C.-H. Jung, and J.-S. Kim, ‘Crystal structure of a minimal nitroreductase, ydjA, from Escherichia coli K12 with and without FMN cofactor.’, *J. Mol. Biol.*, vol. 377, no. 1, pp. 258–67, Mar. 2008, doi: 10.1016/j.jmb.2008.01.004.

[50] M. Blahut *et al.*, ‘Conserved cysteine residues are necessary for nickel-induced allosteric regulation of the metalloregulatory protein YqjI (NfeR) in E. coli.’, *J. Inorg. Biochem.*, vol. 184, pp. 123–133, Jul. 2018, doi: 10.1016/j.jinorgbio.2018.04.016.

[51] J. D. Hillman, ‘Mutant analysis of glyceraldehyde 3-phosphate dehydrogenase in Escherichia coli.’, *Biochem. J.*, vol. 179, no. 1, pp. 99–107, Apr. 1979, doi: 10.1042/bj1790099.

[52] C. Hesslinger, S. A. Fairhurst, and G. Sawers, ‘Novel keto acid formate-lyase and propionate kinase enzymes are components of an anaerobic pathway in Escherichia coli that degrades L-threonine to propionate.’, *Mol. Microbiol.*, vol. 27, no. 2, pp. 477–92, Jan. 1998, doi: 10.1046/j.1365-2958.1998.00696.x.

[53] N. N. Rao, M. F. Roberts, A. Torriani, and J. Yashphe, ‘Effect of glpT and glpD mutations on expression of the phoA gene in Escherichia coli.’, *J. Bacteriol.*, vol. 175, no. 1, pp. 74–9, Jan. 1993, doi: 10.1128/jb.175.1.74-79.1993.

[54] H. G. Ngo, S. G. Mohiuddin, A. Ananda, and M. Orman, ‘Unraveling CRP/cAMP-mediated metabolic regulation in Escherichia coli persister cells’, *Elife*, vol. 13, Jul. 2025, doi: 10.7554/eLife.99735.3.

[55] S. G. Mohiuddin, T. V. Nguyen, and M. A. Orman, ‘Pleiotropic actions of phenothiazine drugs are detrimental to Gram-negative bacterial persister cells’, *Commun. Biol.*, vol. 5, no. 1, p. 217, 2022, doi: 10.1038/s42003-022-03172-8.
